# A detailed study on genetic diversity, antioxidant machinery, and expression profile of drought-responsive genes in rice genotypes exposed to artificial osmotic stress

**DOI:** 10.1038/s41598-023-45661-8

**Published:** 2023-10-26

**Authors:** Bijoya Bhattacharjee, Akib Ali, Krishnappa Rangappa, Burhan U. Choudhury, V. K. Mishra

**Affiliations:** 1 Division of Crop Sciences, ICAR Research Complex for NER, Umiam, Meghalaya 793103 India; 2 Division of System Research and Engineering, ICAR Research Complex for NER, Umiam, Meghalaya 793103 India; 3 ICAR Research Complex for NER, Umiam, Meghalaya 793103 India

**Keywords:** Biotechnology, Molecular biology, Plant sciences

## Abstract

Seasonal variations in rainfall patterns, particularly during sowing, early growing season, and flowering, drastically affect rice production in northeastern India. However, sensitivity to drought stress is genotype-specific. Since 80% of the land in this region is used for rice production, it is crucial to understand how they have adapted to water stress. This study evaluated 112 rice genotypes grown in NE India for seed germination percentage and seedling development under PEG-mediated drought stress. Among the rice genotype, Sahbhagi dhan, RCPL-1-82, Bhalum-3 and RCPL-1-128 showed drought-tolerant traits, while Ketaki Joha, Chakhao, Chandan, RCPL-1-185 and IR-64 were the most drought-sensitive rice genotypes. Drought-tolerant rice also showed significantly higher seed germination potential, proline content, antioxidant activity and expression of drought-responsive genes than drought-sensitive rice genotypes. A similar expression pattern of genes was also observed in the rice genotype treated with a 50% water deficit in pot culture. In addition, drought stress reduced the pollen fertility and yield per plant in sensitive rice genotypes. Molecular markers associated with drought stress were also used to characterize genetic diversity among the rice genotypes studied.

## Introduction

Rice (*Oryza sativa* L.) belongs to the family Poaceae (Gramineae) and is amongst the three strategic crops that form the staple diet of more than half of the world’s population. The global cultivation of rice has been evaluated to be about 850 million tons, and the rice-growing area is estimated to be 256 million hectares. Asia is the leading producer of rice in the world, accounting for around 90% of the world’s crops^1^. Over 75% of global production is consumed by people in Asian countries, and, thus, rice is of great importance to the food security of Asia in precise. Northeast India alone harbours thousands of indigenous rice cultivars/genotypes and is considered the second largest centre of rice origin^2^. Indigenous genotypes are known to constitute a rich beneficial gene pool. Although these genotypes are enriched with nutritive properties, they are susceptible to various biotic and abiotic stresses. In contrast to several other crops, rice is quite vulnerable to dryness during germination, the early stages of seedling growth and flowering stages. Due to the lack of water, severe reductions in seedling germination and growth are seen during drought stress^3^.

Drought causes many physiological and biochemical modifications at levels ranging from sub-cellular to the whole plant. One such modification is the production of reactive oxygen species (ROS), which are harmful to plant metabolism. ROS production is controlled by various enzymatic and non-enzymatic antioxidant defence systems, including catalase (CAT), ascorbate peroxidase (APX), proline oxidase (POX), superoxide dismutase (SOD), monodehydro ascorbate reductase (MDHAR), dehydroascorbate reductase (DHAR), and glutathione reductase (GR), while non-enzymatic catalysts include ascorbate, glutathione, carotenoids, phenolic compounds, proline, glycine betaine, and sugars. Although several QTLs have been characterized for drought tolerance/sensitivity in rice, the expression of a particular gene is genotype and habitat-specific^4–6^. Therefore, recognizing the biochemical mechanisms associated with rice drought stress resistance is still a significant obstacle to the early identification of an effective trait that would aid plant breeders and, in particular, breeding programs. Further, the accumulation of compatible solutes, such as proline and glycine betaine, represents a basic strategy used by plants to protect against osmotic stress; proline is also reported to play a positive role in maintaining membrane integrity under various stresses^7, 8^. Usually, proline accumulation is considered to be higher in stress-tolerant plants than in sensitive plants^9^. Additionally, it is evident that drought influences metabolite levels differently in a genotype-dependent manner. These abiotic stress conditions also influence plant growth patterns/regulations and create disparities in plant mineral nutrition, resulting in secondary consequences.

Several biotechnological tools and techniques have been well established to identify the diversity and crucial mechanism of plant defence against stressors. Molecular markers are useful tools for carefully characterizing and differentiating genotypes and determining their evolutionary relationships as they are rapid, reliable, and require less DNA. Simple Sequence Repeats (SSRs) are highly polymorphic, more reproducible, co-dominant, and dispersed across the genome^10^. A correlation analysis between the genotype segregation via molecular markers with the drought-induced parameters furthermore validates the evolutionary links between potent stress tolerant/sensitive genotypes. Analyses of rice plants in response to drought stress and recognition of drought tolerance mechanisms for the production of drought-adapted crops will potentially result in higher crop yields per unit of water input.

Since the selection of plants under field conditions is tedious due to low heritability, the time required, and the interaction between environmental factors and genotypes, the utility of rice drought-tolerant inside a maintained in vitro condition may be useful to improve the selection efficiency and accuracy in terms of drought tolerance in genotypes which is not affected by environmental factors. Polyethylene glycol (PEG), a non-ionic, non-plasmolyzing and high-molecular-weight compound imitates drought stress in cultured cells similar to that observed in the cells of intact plants affected by drought conditions^11, 12^. Therefore, we used PEG-6000 to create an artificial osmotic stress and its impact on the rice genotype. Further, to check whether the results observed from PEG-mediated drought stress shows similar pattern with that of under the soil culture, we conducted a separate experiment of well-watered and 50% water stress conditions for selected rice genotypes.

Precisely, the objectives of this research were to characterise the effect of artificial drought stress on antioxidant properties and assess the genetic diversity of the local rice genotype grown in Northeast India using drought-linked SSR and RAPD markers to find out potent drought-tolerant genotype. To understand the drought-responsive machinery, we also attempted to profile the expression pattern of several drought-responsive genes in the roots, shoots and young panicles of contrasting drought-tolerant/sensitive rice genotypes.

## Results

### Effect of PEG-mediated drought stress on seed germination, shoot and root length and relative water content

**Figure 1:**
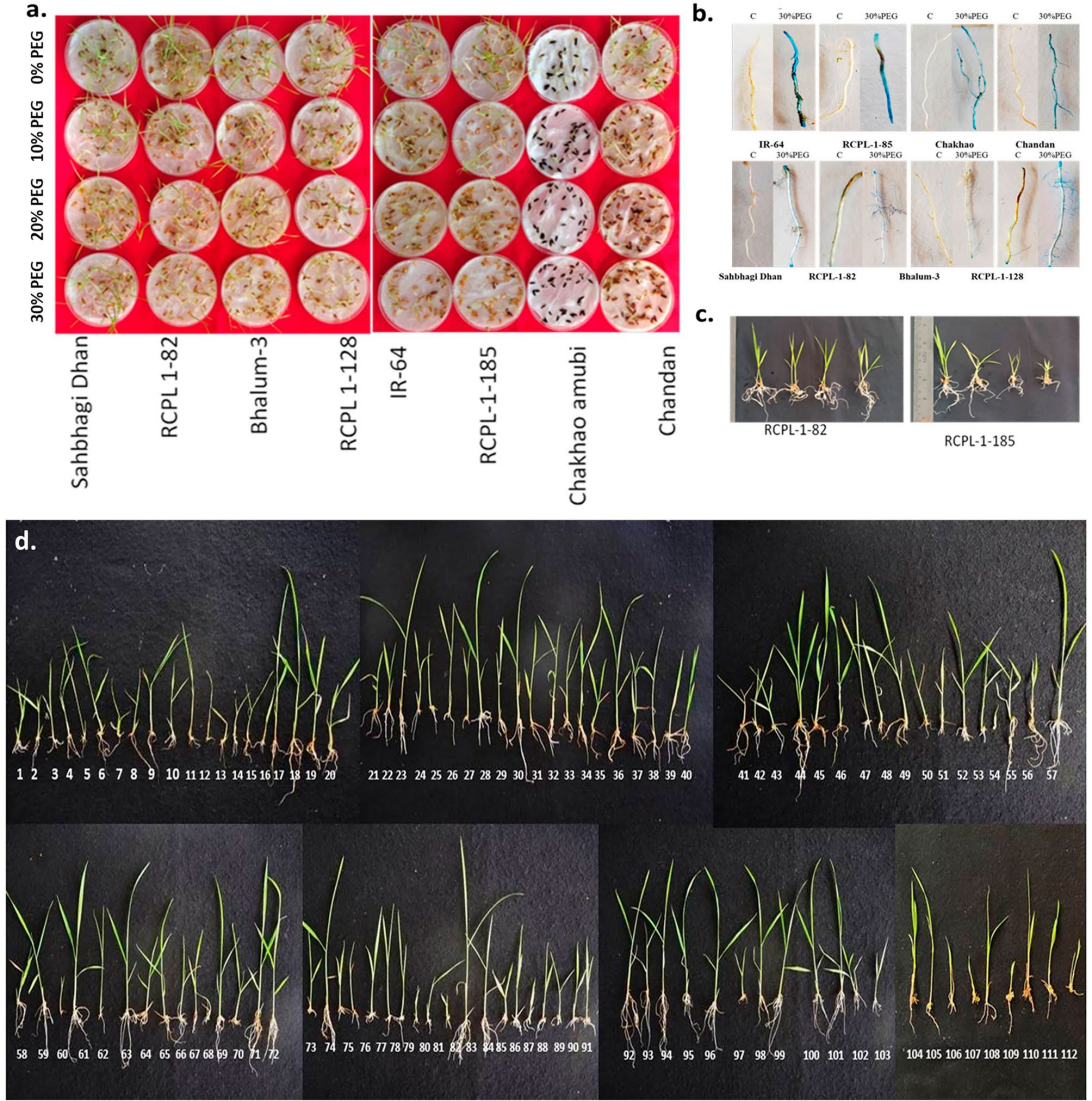
(**a**) A representative picture showing effect of different concentrations of PEG mediated osmotic stress on the seed germination percentage. (**b**) A representative picture of EVANS blue dye staining of the root of rice genotype treated with 30% PEG and control (0% PEG); (**c**) picture showing15 days old rice genotype under different concentrations of PEG-stress; (**d**) picture showing 112 rice genotype grown under 30% PEG; 1. AALIDUMAJU; 2. AJUCENA; 3. ANJALI; 4. SAMBA MAHSURI; 5. BANG; 6. BHALUM 1; 7. BHALUM 2; 8. IR-7277-7-22-1-1; 9. BHALUM 4; 10. IR-78667-1-2-1-1-2; 11. RCPL-1-47; 12. BP-2890-MR8; 13. RCPL-1-13; 14. CHARANGPHOU; 15. RCPL-1-86; 16. COL-4; 17. DAGARDESHI; 18. RCPL-1-101; 19. Fullbadam; 20. GOMTIDHAN; 21. GOVINDOBHOG; 22. Hakuchung; 23. HPR—2558; 24. IORO EPYO; 25. IR-1552; 26. RANJIT; 27. IR-71524-44-1-2-8; 28. BHALUM 3; 29. IR-74052-80-1-1; 30. BHUTMURI; 31. KMP-34; 32. Katak-tara; 33. KASALATH; 34. V-Dhan; 35. KRISHNA; 36. LUNISHREE; 37. MAI-CHING; 38. Megha aromatic; 39. MEGHA RICE 1; 40. MNEO; 41. N-902; 42. NAVEEN; 43. NDR-97; 44. NEPAL RICE; 45. PAIJONG; 46. POKKALI; 47. PR-23079-10; 48. PR-25679-B-9-1; 49. PR-26850-P-J-18-6; 50. PSB-RC2; 51. PURPLE RICE; 52. PYNTHOR; 53. RADHUNIPAGOL; 54. IR64; 55. RCPL-1-102; 56. RCPL-1-108; 57. SAHBHAGI DHAN (IR74371-70-1-1); 58. RCPL-1-113; 59. RCPL-1-100; 60. RCPL-1-117; 61. RCPL-1-104; 62. RCPL-1-105; 63. RCPL-1-107; 64. RCPL-1-109; 65. RCPL-1-110; 66. RCPL-1-115; 67. RCPL-1-115; 68. EPYO; 69. RCPL-1-128; 70. RCPL-1-127; 71. BOG BUTAL; 72. Baglami; 73. RCPL-1-132R; 74. RCPL-1-91; 75. RCPL-1-46; 76. RCPL-1-74; 77. RCPL-1-77; 78. RCPL-1-78; 79. Chandan; 80. RCPL-1-96; 81. RCPL-1-90; 82. RCPL-1-185; 83. RCPL-1-82; 84. RCPL-1-97; 85. RCPL-1-98; 86. SLICKY RICE; 87. Amubi (Chakhao amubi); 88. SANG CHANG; 89. SATABDI; 90. SHASHARANG; 91. SHENGNYA; 92. HANSA; 93. SKAU-390; 94. RCPL-1-112; 94. SUKARDHAN; 96. SUNDARI; 97. SWARNA; 98. TSAMUM FIRRI; 99. TSUMATSUK; 100. UPR-2919; 101. UPR-2992; 102. VANDANA; 103. VL-31331; 104. VIETNAM-3; 105. VIETNAM-1; 106. VL-31329; 107. Ketaki Joha; 108. VPLR-1-7; 109. VR-14; 110. WAB-450-1-1-1-2-P41-HB; 111. YEMSO; 112. ZAM.

Effect of PEG induced drought stress on seed germination percentage revealed significant differences (p < 0.05) between studied rice genotype. As shown in Table 1, while 100% seed germinated in the control (0% PEG), seed germination (%) significantly decreased with further increase in PEGconcentration in the studied rice genotype. Amongst the studied rice genotype Sahbhagi Dhan, RCPL-1-82, Bhalum-3, RCPL 1-128, Baglami and Bhutmari showed comparatively higher seed germination (%) under PEG stress. In contrast Ketaki Joha, Chakhao, Chandan, RCPL-1-185 and IR-64 showed the lowest seed germination (Table 1) under PEG-stress. Although, there was no significant difference in seed germination percentage at different concentrations of PEGtreatment of Sahbhagi Dhan, RCPL-1-82, Bhalum-3 and RCPL-1-128; in contrast, Ketaki Joha, Chakhao amubi, Chandan, RCPL-1-185 and IR-64 seed germination significantly decreased with increase in PEGpercentage (Fig. 1, Table 1). Similarly, root and shoot length (cm) were significantly decreased with increasing PEGconcentrations. Sahbhagi Dhan, RCPL-1-82, Bhalum-3 and RCPL-1-128 also showed higher shoot and root length as compared to Ketaki Joha, Chakhao amubi, Chandan, RCPL-1-185 and IR-64 (Fig. 1). However, shoot to root ratio significantly increased with increase in PEGconcentration in all studied rice genotype except Sahbhagi Dhan, RCPL-1-82, Bhalum-3 and RCPL-1-128. The relative water content studied in the present investigation also revealed similar pattern among different rice genotype (Supplementary table 1). While in control, shoot RWC ranged from 79 to 86%; 70 to 85%, 67 to 84% and 62 to 82% RWC was observed in 10% PEG, 20% PEGand 30% PEGrespectively. In roots, RWC ranged from 72 to 84% in control, 67 to 82% at 10% PEG, 65 to 80% at 20% PEGand 57 to 80% in 30% PEGtreatment. Sahbhagi dhan and RCPL-1-82 showed comparatively high RWC in root and shoots than other genotypes. However, no significant difference of RWC was observed in the Sahbhagi Dhan, RCPL-1-82 at different PEGconcentration. In contrast, RCPL-1-185 and IR-64 showed significant reduction in RWC in both root and shoots. Furthermore, roots of Sahbhagi Dhan, RCPL-1-82, Bhalum-3 and RCPL-1-128 stained light blue as compared to Chakhao amubi, Chandan, RCPL-1-185 and IR-64 under PEG-treatment (Fig. 1). Control devoid of PEG did not change colour after EVANs blue staining.

**Table 1:** Effect of PEG-mediated drought stress on seed germination percentage of 112 rice genotype/lines grown in NE-India; each value is representation of mean ± sd, N = 9 and different letters along the column are statically significant at p < 0.05.

Rice names	Seed germination %			
	0% PEG	10% PEG	20%PEG	30% PEG
AALIDUMAJU	100	82.25 ± 2.19^e^	76.21 ± 2.08^fg^	59.32 ± 1.87^hi^
AJUCENA	100	81.00 ± 2.56^e^	73.96 ± 2.41^fg^	56.11 ± 1.87^h^
ANJALI	100	80.12 ± 2.54^e^	78.95 ± 3.17^g^	65.21 ± 2.17^j^
Baglami	100	89.21 ± 3.11^fg^	87.25 ± 2.37^h^	85.45 ± 2.85^mn^
BANG	100	82.44 ± 2.35^ef^	71.25 ± 2.31^f^	63.93 ± 2.13^ij^
BHALUM 1	100	77.69 ± 2.99^de^	70.25 ± 2.3^f^	62.35 ± 2.08^i^
BHALUM 2	100	81.37 ± 2.56^e^	70.26 ± 3.27^f^	61.95 ± 2.07^i^
BHALUM 3	100	92.01 ± 3.65^g^	89.25 ± 2.3^h^	88.33 ± 2.94^n^
BHALUM 4	100	76.25 ± 2.14^de^	69.54 ± 3.03^ef^	62.15 ± 2.07^i^
BHUTMURI	100	86.24 ± 3.63^f^	85.63 ± 3.06^h^	82.01 ± 2.73^m^
BONG BUTAL	100	84.67 ± 2.14^ef^	83.45 ± 2.3^gh^	82.41 ± 2.75^m^
BP-2890-MR8	100	82.12 ± 2.31^e^	71.25 ± 0.76^f^	62.01 ± 2.07^i^
Chakhao amubi	100	65.23 ± 2.58^bc^	40.23 ± 3.74^b^	20.45 ± 0.68^b^
Chandan	100	56.20 ± 2.91^a^	39.22 ± 4.31^b^	20.15 ± 0.67^b^
CHARANGPHOU	100	79.64 ± 2.34^e^	68.41 ± 2.34^ef^	62.31 ± 2.08^i^
COL-4	100	82.14 ± 1.24^e^	71.27 ± 2.31^f^	63.25 ± 2.11^ij^
DAGARDESHI	100	78.69 ± 3.19^e^	70.25 ± 2.27^e^	62.35 ± 2.08^i^
EPYO	100	82.37 ± 2.14^e^	71.26 ± 2.3^f^	61.25 ± 2.04^i^
Fullbadam	100	76.25 ± 2.98^de^	69.54 ± 2.27^ef^	62.15 ± 2.07^i^
GOMTIDHAN	100	75.46 ± 2.65^d^	68.14 ± 2.2^ef^	61.25 ± 2.04^i^
GOVINDOBHOG	100	72.58 ± 2.55^cd^	65.35 ± 3.07^e^	59.45 ± 1.98^hi^
Hakuchung	100	84.99 ± 2.48^ef^	83.38 ± 2.93^gh^	82.8 ± 2.76^m^
HPR-2558	100	83.42 ± 2.84^ef^	81.20 ± 1.01^g^	79.2 ± 2.64^l^
IORO EPYO	100	63.98 ± 2.84^b^	37.98 ± 3.97^ab^	27.24 ± 0.91^d^
IR-1552	100	54.95 ± 1.78^a^	36.97 ± 2.53^ab^	26.94 ± 0.98^d^
IR64	100	58.21 ± 1.98^ab^	31.41 ± 2.22^a^	14.45 ± 0.48^a^
IR-71524-44-1-2-8	100	80.89 ± 2.54^e^	69.02 ± 2.19^ef^	60.04 ± 2.87^i^
IR-7277-7-22-1-1	100	77.44 ± 2.36^de^	68.00 ± 2.14^ef^	59.14 ± 1.97^hi^
IR74052-80-1-1	100	81.12 ± 2.11^e^	69.01 ± 2.18^ef^	58.04 ± 1.93^hi^
IR-78667-1-2-1-1-2	100	75.00 ± 2.58^d^	67.29 ± 2.14^ef^	58.94 ± 1.96^hi^
KASALATH	100	74.21 ± 3.47^d^	65.89 ± 2.08^e^	58.04 ± 1.93^hi^
Katak tara	100	71.33 ± 3.65^cd^	63.10 ± 0.76^de^	56.24 ± 1.87^h^
Ketaki Joha	100	62.01 ± 2.64^ab^	34.46 ± 2.81^ab^	20.37 ± 0.68^b^
KMP-34	100	82.17 ± 2.77^e^	78.95 ± 2.1^g^	75.99 ± 2.53^l^
KRISHNA	100	79.78 ± 2.14^e^	67.89 ± 0.93^e^	56.66 ± 1.89^h^
LUNISHREE	100	62.89 ± 5.11^b^	36.87 ± 1.92^ab^	25.1 ± 0.84^dc^
MAI-CHING	100	53.86 ± 3.78^a^	35.86 ± 2.98^ab^	24.8 ± 0.73^dc^
Megha aromatic	100	57.12 ± 2.89^ab^	30.30 ± 2.14^a^	26.31 ± 0.88^d^
MEGHA RICE 1	100	79.80 ± 2.37^e^	67.90 ± 2.11^e^	57.9 ± 1.93^h^
MNEO	100	76.35 ± 2.12^de^	66.89 ± 2.07^e^	57 ± 1.90^h^
N-902	100	80.03 ± 2.54^e^	67.90 ± 2.11^e^	55.9 ± 1.86^h^
NAVEEN	100	73.91 ± 2.33^d^	66.18 ± 2.07^e^	56.8 ± 1.89^h^
NDR-97	100	73.12 ± 2.11^d^	64.78 ± 2.01^de^	55.9 ± 1.86^h^
NEPAL RICE	100	70.24 ± 2.54^cd^	61.99 ± 0.68^d^	54.1 ± 1.82^gh^
PAIJONG	100	60.92 ± 2.54^ab^	33.34 ± 2.73^ab^	18.23 ± 2.61^b^
POKKALI	100	81.08 ± 2.36^e^	77.84 ± 2.02^h^	73.85 ± 2.46^kl^
PR-23079-10	100	78.69 ± 2.11^e^	66.77 ± 0.86^e^	54.52 ± 1.82^gh^
PR-25679-B-9-1	100	61.80 ± 2.54^ab^	35.75 ± 3.84^ab^	22.96 ± 0.77^bc^
PR-26850-P-J-18-6	100	52.77 ± 2.23^a^	34.74 ± 2.49^ab^	22.66 ± 0.76^bc^
PS_B_-RC_2_	100	56.03 ± 2.89^ab^	29.18 ± 3.9^a^	24.17 ± 2.24^cd^
PURPLE RICE	100	78.71 ± 2.45^e^	66.79 ± 3.8^e^	55.76 ± 2.61^h^
PYNTHOR	100	75.26 ± 2.85^de^	65.77 ± 2.84^e^	54.86 ± 3.42^gh^
RADHUNIPAGOL	100	78.94 ± 1.56^e^	66.78 ± 2.77^e^	53.76 ± 2.56^gh^
RANJIT	100	72.82 ± 3.11^cd^	65.06 ± 2.76^e^	54.66 ± 2.49^gh^
RCPL-1-102	100	72.03 ± 1.65^c^	63.66 ± 3.92^de^	53.76 ± 2.48^gh^
RCPL-1-108	100	69.15 ± 1.44^c^	60.87 ± 2.77^d^	51.96 ± 3.53^ g^
RCPL-1-112	100	59.83 ± 1.32^ab^	32.23 ± 3.64^ab^	21.09 ± 2.49^b^
RCPL-1-113	100	79.99 ± 1.21^e^	76.72 ± 3.67^fg^	71.71 ± 3.28^k^
RCPL-1-100	100	76.55 ± 2.38^de^	65.75 ± 2.76^e^	53.62 ± 3.33^gh^
RCPL-1-101	100	59.66 ± 1.11^ab^	54.73 ± 0.91^c^	42.06 ± 2.48^f^
RCPL-1-104	100	60.63 ± 1.45^ab^	53.72 ± 0.9^c^	41.76 ± 0.82^ef^
RCPL-1-105	100	63.89 ± 2.14^bc^	58.16 ± 2.77^d^	43.27 ± 0.81^f^
RCPL-1-107	100	76.57 ± 2.64^d^	65.77 ± 2.81^e^	54.86 ± 2.49^gh^
RCPL-1-109	100	73.12 ± 2.01^d^	64.75 ± 2.77^de^	53.96 ± 2.53^gh^
RCPL-1-110	100	76.80 ± 1.58^de^	65.76 ± 2.72^e^	52.86 ± 2.49^gh^
RCPL-1-115	100	70.68 ± 1.54^c^	64.04 ± 2.77^de^	53.76 ± 2.45^gh^
RCPL-1-115	100	69.89 ± 1.36^c^	62.64 ± 2.72^d^	52.86 ± 2.49^gh^
RCPL-1-117	100	78.22 ± 1.24^de^	66.75 ± 2.64^e^	52.83 ± 2.45^gh^
RCPL-1-1-185	100	54.26 ± 1.84^a^	36.25 ± 3.68^ab^	19.56 ± 2.38^b^
RCPL-1-127	100	61.33 ± 1.47^ab^	55.73 ± 3.52^cd^	41.27 ± 3.31^ef^
RCPL-1-128	100	90.75 ± ± 1.56^g^	89.22 ± 1.21^h^	88.00 ± 3.17^mn^
RCPL-1-13	100	68.73 ± 1.32^bc^	61.89 ± 1.2^de^	50.75 ± 1.09^g^
RCPL-1-132R	100	67.93 ± 2.14^bc^	60.49 ± 0.64^d^	49.86 ± 1.08^g^
RCPL-1-46	100	74.49 ± 2.35^d^	66.06 ± 2.67^e^	53.86 ± 0.58^hi^
RCPL-1-46	100	73.69 ± 2.14^d^	64.66 ± 2.63^e^	52.97 ± 2.4^gh^
RCPL-1-74	100	70.82 ± 1.68^c^	61.87 ± 2.58^de^	51.17 ± 2.37^gh^
RCPL-1-77	100	61.50 ± 1.87^ab^	33.22 ± 2.62^ab^	25.29 ± 2.32^cd^
RCPL-1-78	100	81.66 ± 2.99^e^	77.72 ± 2.58^g^	70.92 ± 2.36^k^
RCPL-1-82	100	98.11 ± 1.29^h^	97.54 ± 2.5^i^	96.00 ± 2.32^o^
RCPL-1-86	100	62.30 ± 2.07^ab^	54.72 ± 0.92^c^	40.96 ± 2.25^ef^
RCPL-1-90	100	65.56 ± 2.33^b^	59.16 ± 3.38^d^	42.48 ± 2.81^f^
RCPL-1-91	100	78.24 ± 2.14^de^	66.76 ± 2.52^e^	54.07 ± 3.04^hi^
RCPL-1-96	100	74.79 ± 1.25^d^	65.75 ± 1.11^e^	53.17 ± 2.27^gh^
RCPL-1-97	100	78.46 ± 1.51^de^	66.76 ± 2.1^e^	52.07 ± 1.98^gh^
RCPL-1-98	100	72.35 ± 2.01^c^	65.04 ± 1.17^e^	52.96 ± 0.99^gh^
SAHBHAGI DHAN (IR74371-70-1-1)	100	98.25 ± 1.02^h^	97.10 ± 2.58^i^	96.50 ± 1.05^o^
SAMBA MAHSURI	100	71.59 ± 2.25^c^	64.15 ± 2.53^e^	53.52 ± 2.32^gh^
SANG CHANG	100	70.94 ± 2.24^c^	62.55 ± 2.49^de^	51.62 ± 2.28^g^
SATABDI	100	68.06 ± 2.44^bc^	59.76 ± 2.52^d^	49.82 ± 2.24^g^
SHASHARANG	100	58.74 ± 2.64^ab^	31.11 ± 2.01^a^	23.95 ± 2.27^c^
SHENGNYA	100	78.90 ± 2.33^de^	75.61 ± 2.33^fg^	69.57 ± 1.81^k^
SKAU-390	100	57.52 ± 1.02^ab^	53.71 ± 3.07^c^	41.16 ± 2.1^ef^
SLICKY RICE	100	58.49 ± 2.30^ab^	52.70 ± 2.29^c^	40.86 ± 2.76^ef^
SUKARDHAN	100	61.75 ± 2.65^ab^	57.14 ± 2.23^cd^	42.37 ± 2.06^ef^
SUNDARI	100	74.43 ± 2.44^cd^	64.75 ± 2.22^e^	53.96 ± 2.01^gh^
SWARNA	100	70.98 ± 2.87^c^	63.73 ± 3.17^de^	53.06 ± 2.45^gh^
TSAMUM FIRRI	100	74.66 ± 2.36^d^	64.74 ± 2.22^e^	51.96 ± 2.85^g^
TSUMATSUK	100	68.54 ± 2.11^bc^	63.02 ± 2.94^e^	52.86 ± 2.89^gh^
UPR-2919	100	67.75 ± 1.56^bc^	61.62 ± 2.96^de^	51.96 ± 2.65^g^
UPR-2992	100	70.87 ± 2.14^c^	62.91 ± 2.22^de^	51.65 ± 2.66^g^
VANDANA	100	70.07 ± 2.32^c^	61.51 ± 0.73^de^	50.76 ± 2.88^g^
V-Dhan	100	67.20 ± 1.24^bc^	58.72 ± 1.72^d^	48.96 ± 1.66^g^
VIETNAM-3	100	57.88 ± 1.21^ab^	30.07 ± 2.23^a^	23.08 ± 0.65^c^
VIETNAM-1	100	78.04 ± 1.45^de^	74.57 ± 2.27^fg^	68.71 ± 2.01^k^
VL-31329	100	74.60 ± 1.11^d^	63.60 ± 2.23^de^	50.62 ± 2.04^g^
VL-31331	100	57.71 ± 1.51^ab^	52.58 ± 2.2^c^	39.06 ± 2.01^e^
VPLR-1-7	100	58.68 ± 1.02^ab^	51.57 ± 2.4^c^	38.75 ± 1.98^e^
VR-14	100	61.94 ± 1.24^ab^	56.01 ± 2.5^d^	40.27 ± 2.58^ef^
WAB-450-1-1-1-2-P_41_-HB	100	74.62 ± 1.23^d^	63.61 ± 2.13^de^	51.86 ± 1.98^g^
YEMSO	100	71.17 ± 2.21^c^	62.60 ± 2.97^de^	50.96 ± 1.92^g^
ZAM	100	74.84 ± 1.25^d^	76.21 ± 2.08^g^	49.86 ± 2.67^g^

### Diversity analysis of rice genotype using SSR and RAPD marker

**Figure 2:**
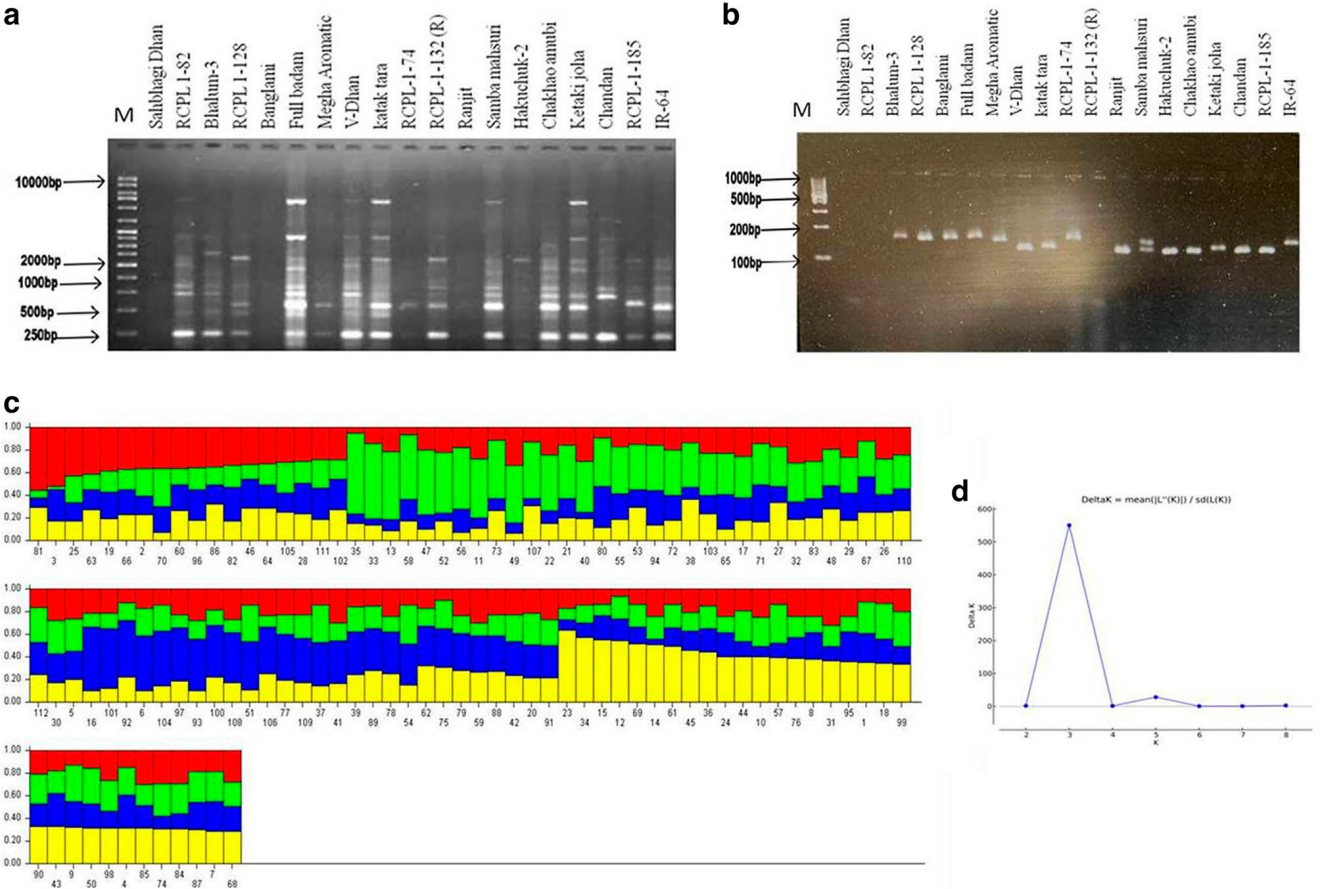
A representative electrophoresis gel pic showing amplified DNA bands in 19 rice genotype using RAPD maker (**a**) and SSR marker (**b**); barplot (**c**) of 112 rice genotype developed based on the presence or absence of bands (SSR and RAPD markers) using STRUCTURE software, similar colour indicates genetic similarity between the rice genotype; A line graph depicting K value of the population study (**d**).

Preliminary genetic relationship among 112 studied rice genotype was assessed with 57 previously reported drought stress tolerance linked Simple Sequence Repeat (SSR) and 5 Random Amplified Polymorphic DNA (RAPD) markers. A total of 1395 alleles were scored using SSR and RAPD marker. Out of which 219 (15.7%) were monomorphic and 1176 (84.3%) were polymorphic. A representing banding pattern of studied rice genotypes were shown in Fig. 2. A summary of the molecular markers used in the present study were shown in the Supplementary Table 2. In RAPD, OPB-10 showed highest range of allele size; while in SSR marker, RM3 shows more polymorphism and maximum number of alleles as compared to other primer and RM11 generated least number of alleles (Fig. 2). RM3233 showed highest range of allele size (165–269 bp). The allele frequency ranged from 0.25 to 0.71 with an average value of 0.46. The polymorphism information content (PIC) ranged from 0.48 to 0.93 averaging 0.77 (Supplementary Table 2). While RM3 showed the highest PIC value, RM204 showed the lowest PIC value, which indicates the potential use of these markers in genotyping and diversity analysis study. The model-based population structure analysis performed with STRUCTURE software revealed highest K value of 3 (Fig. 2). A phylogenetic tree generated using NTSYSpc 2.10 based on similarity index SM coefficient divided the studied rice genotype into two clades (Supplementary Fig. 1). While clade-I is further dived into three clusters. Within Cluster-I, Sahbhagi Dhan, Bhalum-3, RCPL-1-82, Banglami and V-Dhan which showed high seed germination percentage and RWC formed one sub-group; In cluster-II, Full badam, Ketaki joha and RCPL-1-185 which also showed high RWC and seed germination % formed one group. On the other hand, Clade-II includes RCPL-1-128, chandan and IR-64 which showed drought-sensitive characteristics. The population of the 112 investigated rice genotype was found to be genetically diverse from each other.

Based on the physio-morphological traits and genetic diversity analysis, we further selected four rice genotypes showing two contrasting characters i.e., putative drought-tolerant (Sahbhagi Dhan and RCPL-1-82) and putative drought-sensitive (RCPL-1-185 and IR-64) for antioxidant profiling and molecular profiling of drought-sensitive genes.

### Effect of PEG-stress on proline, H_2_O_2_, AaS, DHA and total ascorbate content in drought-tolerant/sensitive rice

As shown in Table 2, drought-tolerant and sensitive rice genotype showed significant differences in the accumulation of proline, H_2_O_2_, AaS content, DHA content and total ascorbate in shoots as well as roots on 15th days after PEG treatment. While accumulation of proline and H_2_O_2_ in the roots and shoot of Sahbhagi Dhan and RCPL-1-82 enhanced with increase in PEG concentration, the same showed highest accumulation at 10% PEG treatment in roots and shoots of RCPL-1-185 and IR-64, thereafter declined with further increase in PEG concentration. Sahbhagi Dhan and RCPL-1-82 showed highest abundance of proline at 30% PEG treatment, while IR-64 and RCPL-1-185 showed highest proline content at 10% PEG treatment. Similarly, Sahbhagi Dhan and RCPL-1-82 also showed significantly higher accumulation of H_2_O_2_ as compared to RCPL-1-185 and IR-64. Unlike proline and H_2_O_2_ content, while AaS content declined with increasing PEG concentration, DHA content and total ascorbate increased in the roots and shoot tissues of all studied rice genotype at 0% to 30% PEG treatment. However, there were significant differences between the drought-tolerant (Sahbhagi Dhan and RCPL-1-82) and drought-sensitive (RCPL-1-185 and IR-64) rice genotypes.

**Table 2:** Effect of PEG mediated drought stress on proline content (µmol g^−1^ dry weight), H_2_O_2_ (µmol g^−1^ fresh weight), AaS content (nmol g^−1^ fresh weight), DHA content (nmol g^−1^ fresh weight) and total ascorbate (nmol g^−1^ fresh weight).

	PEG treatment	Shoot tissues	Root tissues						
		Sahbhagi Dhan	RCPL 1-82	RCPL-1-185	IR-64	Sahbhagi Dhan	RCPL 1-82	RCPL-1-185	IR-64
Proline content (µmol g^−1^ dry weight)	0% PEG	31.78 ± 1.07^de^	32.74 ± 1.10^d^	26.65 ± 0.90^c^	22.14 ± 0.75^b^	35.14 ± 1.18^e^	28.13 ± .95^c^	23.41 ± 0.79^b^	19.54 ± 0.66^a^
	10% PEG	89.54 ± 2.57^e^	81.41 ± 2.34^d^	64.49 ± 1.36^c^	61.43 ± 0.9^b^	87.63 ± 1.94^e^	69.11 ± 1.88^d^	63.12 ± 1.87^bc^	55.77 ± 1.89^a^
	20%PEG	171.12 ± 3.23^e^	167.25 ± 3.16^e^	57.63 ± 1.66^b^	59.11 ± 1.31^b^	115.24 ± 2.18^d^	102.65 ± 1.94^c^	58.47 ± 1.29^b^	52.35 ± 1.18^a^
	30% PEG	227.36 ± 5.84^e^	224.45 ± 5.77^e^	47.41 ± 3.02^a^	45.39 ± 2.97^a^	170.35 ± 4.38^d^	159.35 ± 4.1^c^	52.45 ± 3.40^b^	41.36 ± 3.12^a^
H_2_O_2_ (µmol g^−1^ fresh weight)	0% PEG	5.18 ± 0.26^c^	5.27 ± 0.26^c^	4.67 ± 0.23^b^	4.21 ± 0.43^a^	5.51 ± 0.56^c^	4.81 ± 0.47^b^	4.34 ± 0.44^a^	3.95 ± 0.4^a^
	10% PEG	11.45 ± 0.57^c^	10.64 ± 0.53^c^	8.95 ± 0.45^ab^	8.64 ± 0.43^ab^	11.26 ± 0.56^c^	9.41 ± 0.47^b^	8.81 ± 0.44^ab^	8.08 ± 0.40^a^
	20%PEG	19.81 ± 0.99^d^	19.43 ± 0.97^d^	8.46 ± 0.42^ab^	8.61 ± 0.43^b^	14.22 ± 0.71c	12.97 ± 0.65^c^	8.55 ± 0.43^ab^	7.94 ± 0.40^a^
	30% PEG	25.64 ± 1.28^d^	25.35 ± 1.27^d^	7.64 ± 0.38^a^	7.44 ± 0.37^a^	19.94 ± 1.0^c^	18.84 ± 0.94^c^	8.15 ± 0.41^b^	7.04 ± 0.33^a^
AaS content (nmol g^−1^ fresh weight)	0% PEG	47.74 ± 2.39^c^	45.45 ± 2.27^c^	31.45 ± 1.57^a^	31.14 ± 1.56^a^	42.04 ± 2.10^c^	40.94 ± 2.05^c^	31.31 ± 1.57^a^	30.58 ± 1.53^a^
	10% PEG	42.11 ± 2.32^d^	40.73 ± 2.24^d^	27.76 ± 1.53	25.91 ± 1.43^b^	36.52 ± 1.83^c^	35.27 ± 1.76^c^	24.85 ± 1.24^ab^	22.24 ± 1.11^a^
	20%PEG	35.95 ± 1.62^d^	33.14 ± 1.49^ cd^	20.74 ± 0.93^b^	19.54 ± 0.88^ab^	33.76 ± 1.69^ cd^	31.91 ± 1.60^c^	20.25 ± 1.01^ab^	18.14 ± 0.91^a^
	30% PEG	21.18 ± 1.12^e^	19.97 ± 1.06^de^	7.67 ± 0.41^c^	5.21 ± 0.28^a^	18.51 ± 0.93^d^	17.81 ± 0.89^d^	5.34 ± 0.27^a^	5.95 ± 0.30^b^
DHA content (nmol g^−1^ fresh weight)	0% PEG	48.08 ± 2.40f.	49.54 ± 2.48f.	32.21 ± 1.64^b^	29.09 ± 1.45^a^	50.18 ± 2.51f.	43.80 ± 2.19^e^	39.50 ± 1.98^d^	35.98 ± 1.08^c^
	10% PEG	68.94 ± 3.79^d^	60.71 ± 3.04^d^	43.15 ± 2.07^b^	38.74 ± 2.13^a^	74.12 ± 2.02^e^	64.03 ± 3.12^ cd^	54.12 ± 2.98^c^	52.03 ± 2.86^c^
	20%PEG	95.62 ± 4.30^de^	92.15 ± 4.15^de^	59.33 ± 2.67^b^	54.01 ± 2.43^a^	89.44 ± 4.02^de^	81.98 ± 3.69^d^	67.78 ± 3.05^c^	62.21 ± 2.80^bc^
	30% PEG	111.36 ± 5.90^e^	108.45 ± 5.75^de^	79.49 ± 4.21^b^	76.43 ± 4.05^ab^	101.41 ± 5.37^d^	92.40 ± 4.90^c^	71.19 ± 3.77^a^	70.50 ± 3.74^a^
Total ascorbate (nmol g^−1^ fresh weight)	0% PEG	95.82 ± 4.79^d^	94.99 ± 4.75^d^	63.66 ± 3.18^b^	60.23 ± 3.01^a^	92.21 ± 4.61^d^	84.73 ± 1.24^c^	70.82 ± 3.54^b^	66.56 ± 3.33^ab^
	10% PEG	111.05 ± 6.11^e^	101.44 ± 5.58^de^	70.91 ± 3.90^b^	64.65 ± 3.56^a^	110.64 ± 5.90^e^	99.29 ± 5.28^d^	78.97 ± 4.22^b^	74.26 ± 3.97^bc^
	20%PEG	131.57 ± 2.92f.	125.29 ± 5.64^de^	80.07 ± 3.60^b^	73.55 ± 3.31^a^	123.20 ± 5.71^de^	113.89 ± 5.28^d^	88.02 ± 4.06^c^	80.34 ± 3.71^b^
	30% PEG	132.54 ± 4.02^e^	128.42 ± 3.81^e^	87.16 ± 4.62^b^	81.64 ± 4.33^a^	119.92 ± 6.30^d^	110.21 ± 5.79^c^	76.53 ± 4.04^a^	76.45 ± 4.03^a^

Each value is representation of mean ± sd, N = 9 and different letters along the row are statically significant at p < 0.05.

### Antioxidant enzyme activity in drought-tolerant and sensitive rice under PEG-stress

**Figure 3:**
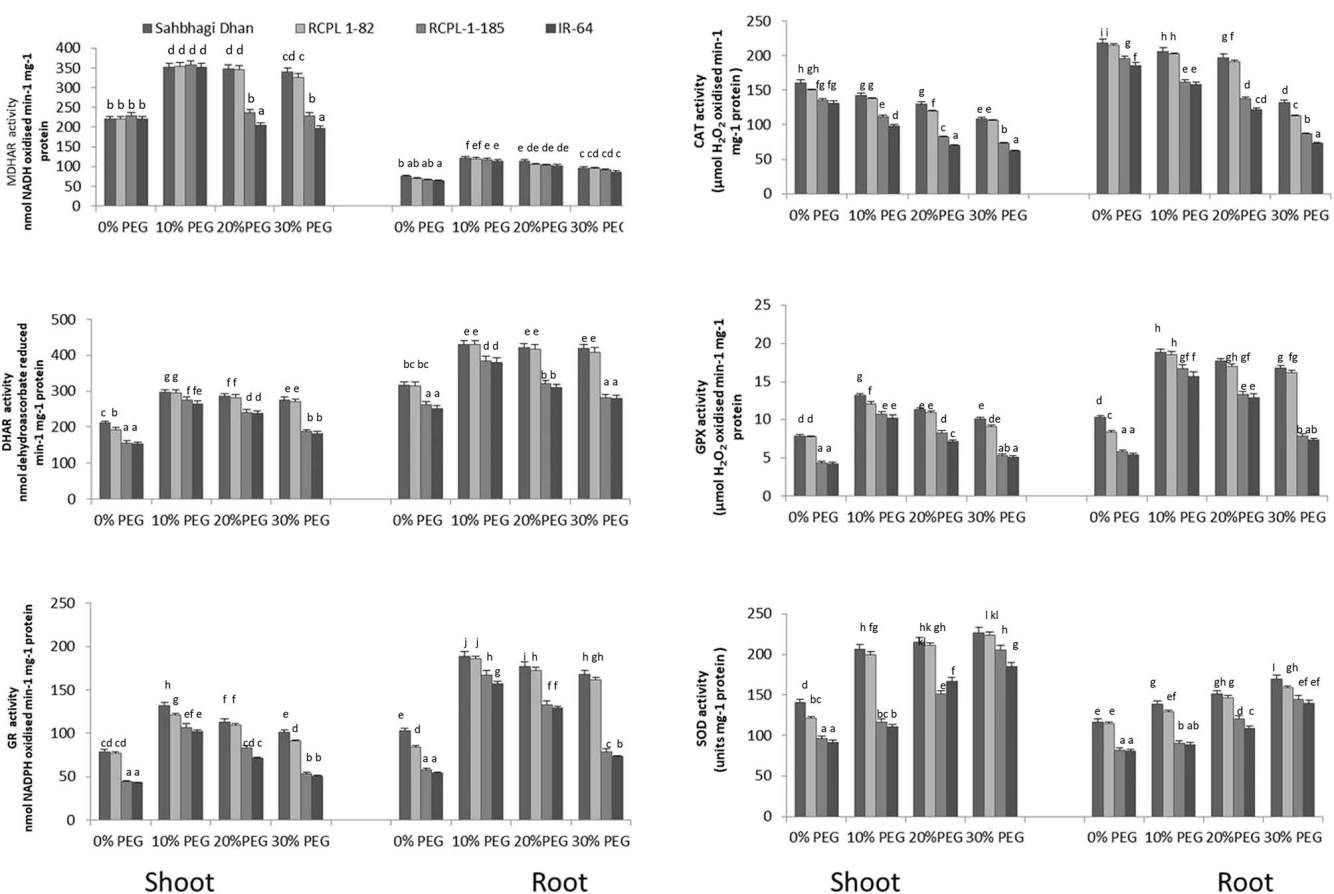
Graphical representation of MDHAR, CAT, DHAR, GPX, GR and SOD activity in the shoot and root tissues of Sahbhagi Dhan, RCPL-1-82, RCPL-1-185 and IR-64 under PEG-mediated drought stress on 15th day after treatment. Each value is representation of mean ± sd, N = 9 and different letters indicates statically significant at p < 0.05.

To decipher the comparative estimation of redox potential of antioxidants between drought-tolerant and sensitive rice genotypes, we performed antioxidant enzyme activity using six different enzyme assays viz. MDHAR, DHAR, GPX, GR, SOD and CAT activity assay. As shown in Fig. 3, antioxidant enzyme activity assays of MDHAR, DHAR, GPX and GR revealed a similar pattern of enzyme activity in the rice genotype treated with different concentration of PEG. In all four assays, enzyme activity was found higher at 10% PEG treatment as compared to control. However, enzyme activity declined with further increase in PEG concentration (10% to 30%) in the root and shoots of all four rice genotype studied. The rate of declining enzyme activity in drought-sensitive rice genotypes (RCPL-1-185 and IR-64) was much higher as compared to drought-tolerant rice genotype (Sahbhagi Dhan and RCPL-1-82). Whereas SOD activity increased with increase in PEG concentration in the root and shoot of all four rice genotype, CAT activity decreased from control to 30% PEG treated plants. While shoot showed significantly higher SOD and MDHAR activity, root showed significantly higher DHAR, GR, CAT and GPX activity. Additionally, Sahbhagi Dhan and RCPL-1-82 showed significantly much higher antioxidant activity in both roots and shoots in all PEG treatments as compared to RCPL-1-185 and IR-64.

### Identification and expression pattern of drought-responsive genes in drought-tolerant/sensitive rice genotypes under PEG-stress

**Figure 4:**
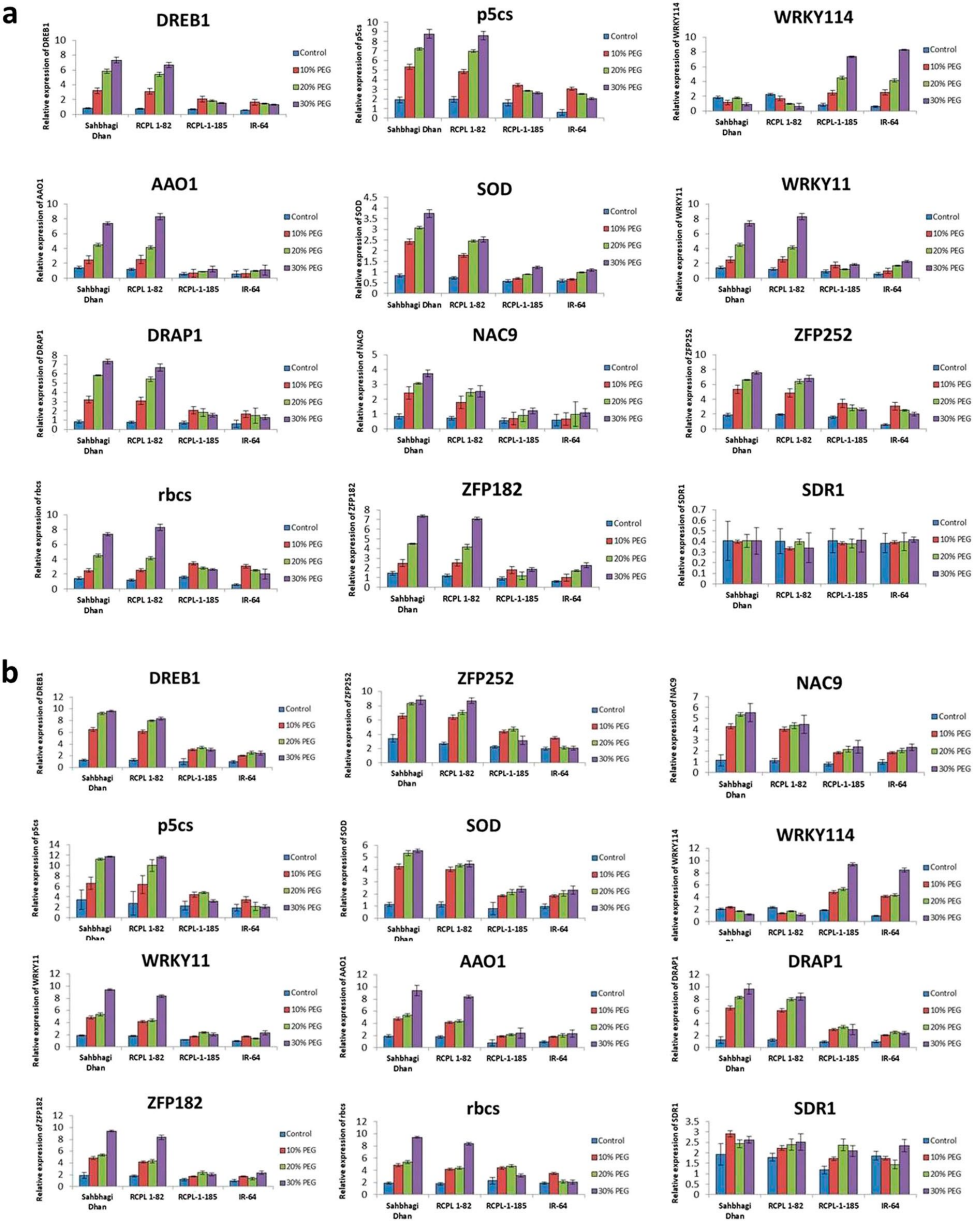
Graphical representation of expression profile of *DREB1*, *p5cs*, *rbcs*, *AAO1*, *SOD*, *WRKY11*, *WRKY114*, *SDR1*, *NAC9*, *ZFP252*, *ZFP182* and *DRAP1* genes in shoots (**a**) and roots (**b**) of Sahbhagi Dhan, RCPL-1-82, RCPL-1-185 and IR-64 under control (C) and PEG-mediated drought stress (T) on 15th day after treatment. Each value is representation of mean ± sd, N = 9 and statically significant at p < 0.05.

To understand the molecular mechanism underlying drought sensitivity in drought-tolerant/sensitive rice, we first identified 12 candidate reference genes viz. *DREB1*, *p5cs*, *rbcs*, *AAO1*, *SOD*, *WRKY11*, *WRKY114*, *SDR1*, *NAC9*, *ZFP252*, *ZFP182* and *DRAP1* which are directly or indirectly linked to drought stress based on available literature. The expression profile of these genes showed a significant difference between the tolerant rice genotype and the sensitive rice genotype at different PEG concentrations (Fig. 4). Expressions of *DREB1*, *p5cs*, *SOD* and *WRKY11* were significantly induced with increasing PEG treatment in the roots and shoots of Sahbhagi Dhan and RCPL-1-82. In contrast, expression of *DREB1* and *p5cs* showed the highest at 10% and 20% PEG respectively in the shoot and roots of RCPL-1-185 and IR-64, thereafter declined in expression. While expression of *SOD* and *WRKY11* increased with increasing PEG concentration in root and shoots of Sahbhagi Dhan and RCPL-1-82. However, it was induced weakly by the PEG-mediated drought stress in RCPL-1-185 and IR-64. Although there was no significant difference in expression of *DREB1*, *p5cs, SOD* and *WRKY11* between RCPL-1-185 and IR-64, it is clearly visible that *DREB1*, *p5cs SOD* and *WRKY11* expressed much higher in Sahbhagi Dhan and RCPL-1-82 as compared to RCPL-1-185 and IR-64. Similarly, while expression of *WRKY114* decreased in the tolerant rice line, it was induced with increasing PEG stress in the sensitive rice line. On the other, we did not find a significant difference in the expression of *SDR1* in both root and shoot tissues of treated and control plants. While the expression of NAC9, ZFP252, ZFP182, and DRAP1 was upregulated with increasing PEG-mediated drought stress in Sahbhagi Dhan and RCPL1-82, these genes showed downregulation of expression in RCPL-1-185 and IR-64.

### Physio-molecular response of rice under drought stress in pot culture during seedling stage

**Figure 5:**
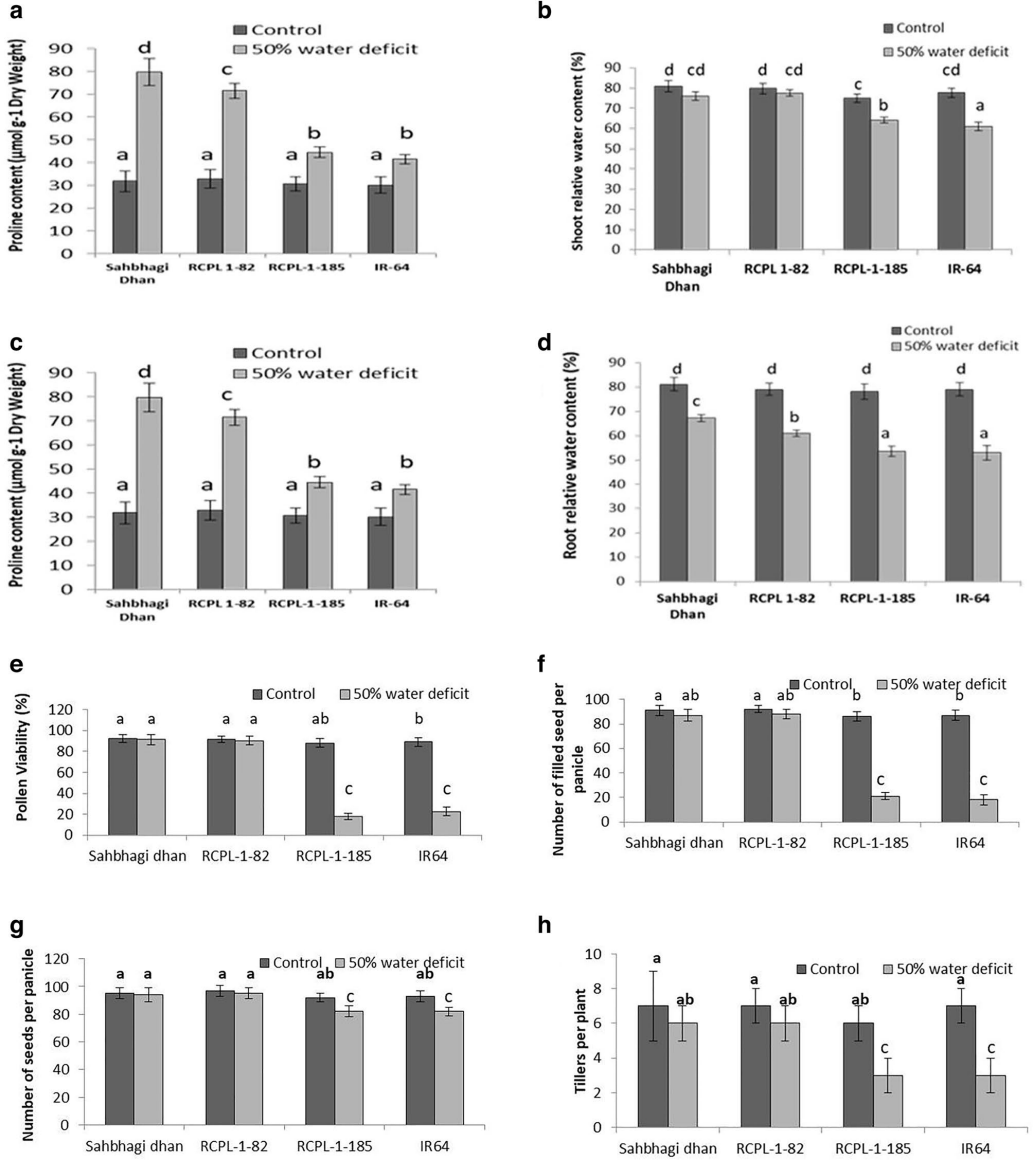
Graphical representation of proline content in the shoot (**a**), proline content in the root (**c**), shoot relative water content (**b**), root relative water content (**d**), % pollen viability (**e**), total number of filled seeds per plant (**f**), total number of seeds per panicle (**g**); tillers per plant (**h**) in Sahbhagi dhan, RCPL-1-82, IR64 and RCPL-1-185 maintained at control (well watered) and 50% water deficit condition. Each value is representation of mean ± sd, N = 9 and different letter indicates statically significant at p < 0.05.

An experiment was conducted on pot culture to check if the result acquired from PEG-mediated drought stress correlates with the drought stress under pot (soil) culture. Four rice genotypes namely Sahbhagi Dhan, RCPL 1-82, RCPL-1-185 and IR-64 were subjected to drought stress with two treatments namely Well-watered (Control; 100% water available) and 50% water deficit treated (Supplementary Fig. 2). The result revealed that RWC significantly reduced in root and shoots of RCPL-1-185 and IR-64 under drought as compared to control. Proline content significantly increased in Sahbhagi Dhan and RCPL-1-82 as compared to RCPL-1-185 and IR-64 under drought stress (Fig. 5).

**Figure 6:**
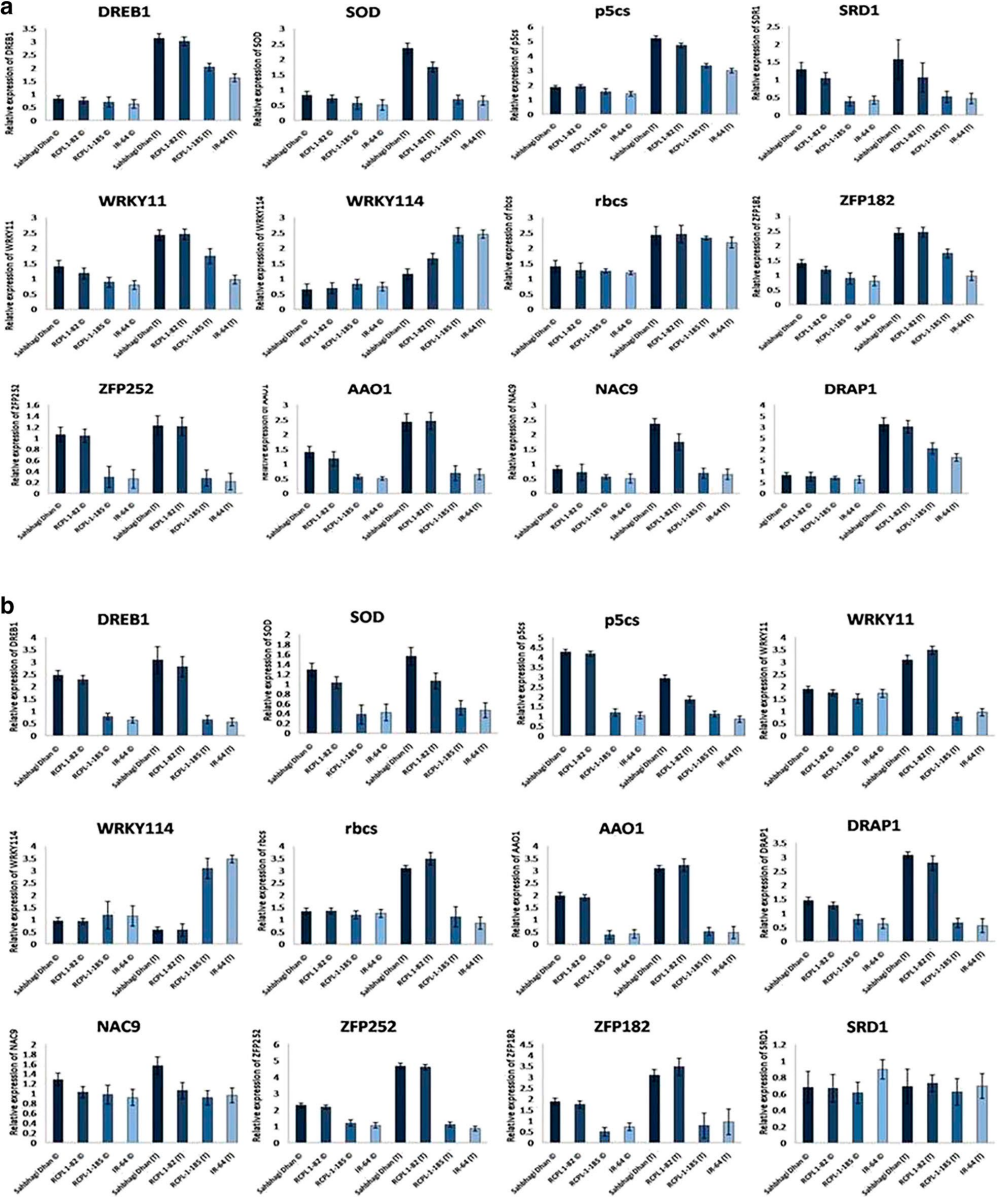
Graphical representation of relative expression of *DREB1*, *p5cs*, *rbcs*, *AAO1*, *SOD*, *WRKY11*, *WRKY114*, *SDR1*, *NAC9*, *ZFP252*, *ZFP182* and *DRAP1* genes in shoots (**a**) and roots (**b**) of Sahbhagi Dhan, RCPL-1-82, RCPL-1-185 and IR-64 under control (well watered) and 50% water deficit condition. Each value is representation of mean ± sd, N = 9 and statically significant at p < 0.05.

Furthermore, the expression of selected drought-responsive genes such as *DREB1, p5cs, rbcs, AAO1, SOD, WRKY11, WRKY114, SDR1, NAC9, ZFP252, ZFP182* and *DRAP1* in the root and shoot tissues of Sahbhagi Dhan, RCPL-1-82 RCPL-1-185 and IR-64 also revealed similar pattern of expression as shown under PEG-mediated stress. While *DREB1, p5cs, rbcs, AAO1, SOD, WRKY11, NAC9, ZFP252, ZFP182* and *DRAP1* upregulated in the root and shoot tissues of Sahbhagi Dhan and RCPL-1-82, *WRKY114* gene showed high expression in root and shoot of RCPL-1-185 and IR-64 under drought stress (Fig. 6).

### Effect of drought stress on pollen fertility, rice yield and expression of drought-responsive gene under pot culture

Pollen fertility is an important aspect in screening potent drought-tolerant and sensitive genotypes as it directly impacts seed setting and grain yield. The microscopic analysis of pollens stained with Potassium Idode stain (I_2_-KI) revealed a significant reduction in the pollen fertility percentage in drought-stressed RCPL-1-185 and IR-64 than in control (Fig. 5). No significant differences were observed in Sahbhagi Dhan and RCPL-1-82. As shown in Supplementary Fig. 6, Sahbhagi Dhan and RCPL-1-82 showed round-shaped, dark-coloured pollen under both control and drought stress, while RCPL-1-185 and IR-64 showed very light-coloured and broken pollen compared to control. Additionally, the number of tillers and filled grains decreased in drought-stressed RCPL-1-185 and IR-64 as compared to the control. While, less than a 5% decrease in filled grains was observed in Sahbhagi Dhan and RCPL-1-82, in contrast, a > 75% decrease in filled grains per panicle was observed in RCPL-1-185 and IR-64 (Fig. 5).

**Figure 7:**
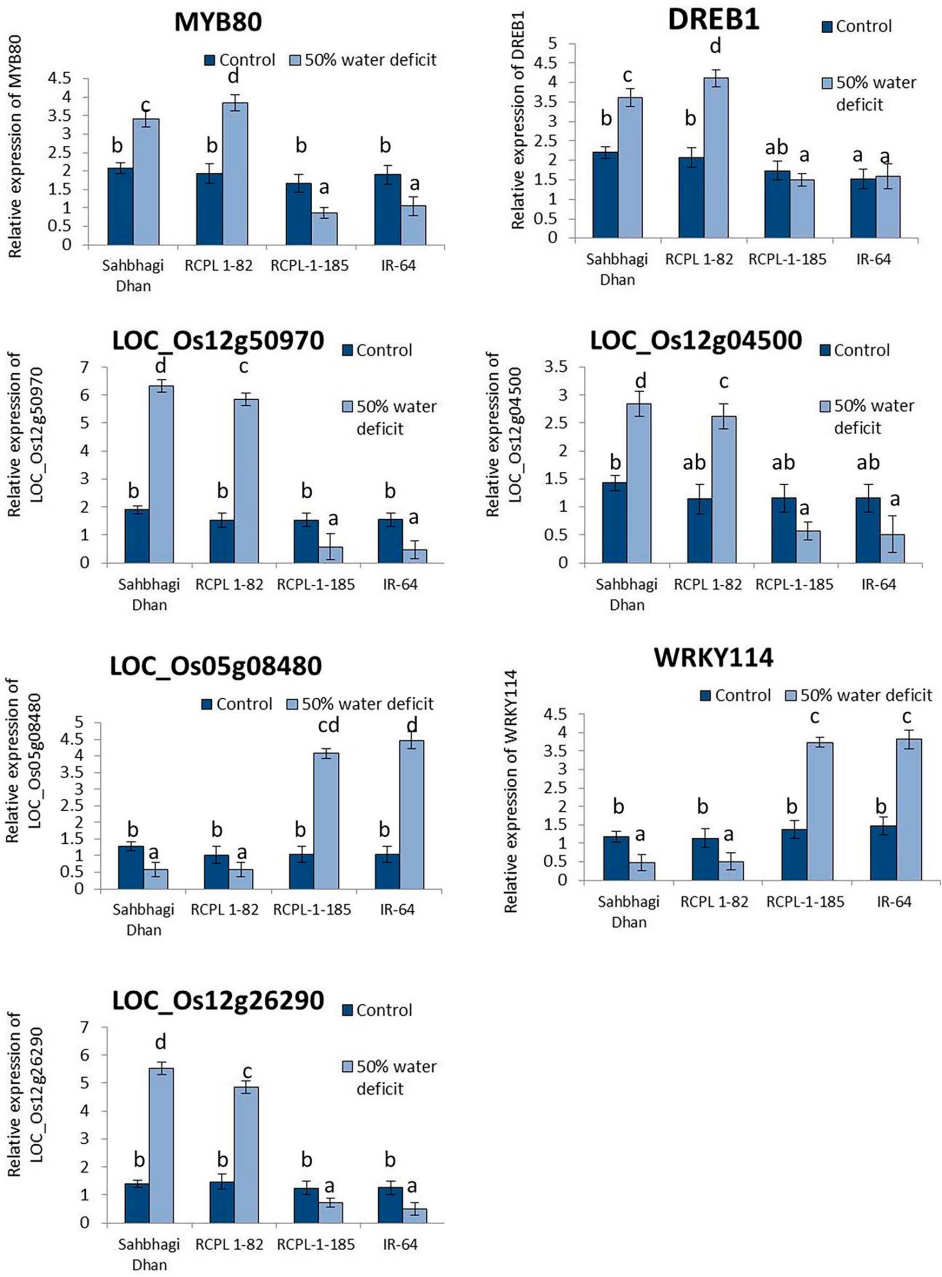
Graphical representation of relative expression of *DREB1, LOC_Os12g04500*, *LOC_Os02g50970*, *LOC_Os12g26290*, *LOC_Os05g08480*, *MYB80* and *WRKY114* genes in the young panicle of Sahbhagi Dhan, RCPL-1-82, RCPL-1-185 and IR-64 under control (well watered) and 50% water deficit condition. Each value is representation of mean ± sd, N = 9 and different letter indicates statically significant at p < 0.05.

Further, to check whether the pollen fertility and grain yield correlate with the expression of previously identified drought-responsive genes between different rice genotypes, we checked transcript abundance of *DREB1*, *LOC_Os12g04500*, *LOC_Os02g50970*, *LOC_Os12g26290*, *LOC_Os05g08480*, *MYB80* and *WRKY114* genes in the young panicle (Fig. 7). The results revealed significant reduction in expression of *LOC_Os12g04500*, *LOC_Os02g50970*, *LOC_Os12g26290* and *MYB80* genes in treated RCPL-1-185 and IR-64 (drought-sensitive) than in Sahbhagi Dhan and RCPL-1-82 (drought-tolerant). While *LOC_Os05g08480* and *WRKY114* genes are upregulated in the young panicle of RCPL-1-185 and IR-64 under drought stress.

## Discussion

Abiotic stresses like droughts drastically affect crop performance by impairing growth, development, and productivity. However, the most effective way to alleviate drought stress in plants is by breeding drought-tolerant cultivars. In the past few decades, rice breeders have developed many drought-tolerant rice genotypes to improve the plant’s resilience under drought conditions^13^. However, the effectiveness of any transgenic/hybrid variety depends on the adaptability to a particular habitat or environment. The primary way to achieve such a goal is by screening germplasm in the field and as well as greenhouse conditions. Since multiple factors affect the performance of a crop in the field condition, therefore, a well-maintained in-house condition may be efficient in finding better experimental results. Although drought affects the life cycle of any plant; seed germination stages, seedling stages and reproductive stages are the most critical stages that determine the growth and yield respectively^14, 15^. According to the Indian Meteorological Department of India (IMD), NE-India has received 31% less rainfall since 2018. NE-India has also been witnessing a series of weather anomalies, which have directly affected the major staple crop of this region i.e., rice. Therefore, in this study, 112 rice genotypes grown in NE-India were taken into consideration for a detailed analysis against PEG-mediated drought stress and further validated with the field/pot culture study. The primary goal of this study was to identify some potent drought-tolerant cultivars specific to NE-India and analyse of detailed mechanism underlying drought tolerance traits. We included two rice genotypes developed by the International Rice Research Institute (IRRI) namely Sahbhagi Dhan as a positive control (drought-tolerant released genotype) and IR-64 as a negative control (drought-sensitive genotype). Our preliminary study on the effect of PEG-mediated drought stress on seed germination and relative water content showed Sahbhagi Dhan, RCPL-1-82, Bhalum-3, RCPL-1-128, Baglami and Bhutmari as potent drought-tolerant rice genotype. The increasing PEG concentration did have much impact on germination and RWC on these plants as compared to Ketaki Joha, Chakhao, Chandan, RCPL-1-185 and IR-64. These variations in phenotypic and physiological characteristics could be attributed to genotype-specific stress tolerance mechanisms^15^. Evan’s dye staining of the roots of the selected rice line confirmed the enhanced accumulation of antioxidants in the tolerant rice genotype as compared to the sensitive genotype.

Our study on molecular profiling using drought stress-linked markers for the studied genotypes showed a high degree of diversification. The identified allelic variants in the form of amplified product size (molecular weight) for each SSR and RAPD marker were documented to find out the allele mining set for the linked markers of the studied genotypes in relation to drought stress tolerance. Since SSR markers are abundant in the rice genome, co-dominance, and have a high polymorphism rate, they are widely utilised in rice genetics^16^. Although RAPD markers are less reproducible; however, RAPD technology still has several key benefits, including its suitability for work on anonymous genomes, applicability to small amounts of DNA, and low cost^17^. Therefore, we selected more SSR markers^18–20^ and only a few RAPD markers^21, 22^ to check if they would produce some significant results. The result revealed that RAPD marker OPB-10 and SSR marker RM3, RM3233 were the best-suited markers to check genetic diversities under PEG-mediated drought stress in rice as these markers showed the highest range of polymorphisms and maximum number of alleles as compared to other primers. It was interesting to see that most of the rice genotypes that showed drought-tolerant/sensitivity using phenotyping and physiological screening also formed separate groups in the phylogenetic tree generated via NTSYSpc-based SSR and RAPD marker. These preliminary results indicate a possible genetic linkage between the genotypes. Since the markers used are linked to drought/water stress-related genes, the result could indicate that each group may share common alleles or haplotypes of the water stress–related genes involved. However, further research is needed to establish a proper linkage between the genotypes. Based on the cumulative results and their sensitivity to drought stress, studied rice genotypes can be categorized into three groups such as tolerant (Group I), moderately sensitive (Group II) and highly sensitive (Group III). While Group I includes Sahbhagi Dhan, RCPL-1-82, Bhalum-3, RCPL-1-128, Group III includes Ketaki Joha, Chakhao amubi, Chandan, RCPL-1-185 and IR-64 and group-II includes the rest of the studied genotype (Supplementary Table 5). Our results showed a higher drought-tolerance capacity of RCPL-1-82 apart from Sahbhagi Dhan and Baglami. Our results of sensitive rice are in conformity with the Sahoo et al.^23^. They reported Ketaki joha and Chandan as among the sensitive rice genotypes. However, we also found that IR-64 and RCPL-1-185 were more drought-sensitive than Ketaki joha and Chandan. We observed purple rice var. Chakhao amubi is moderately sensitive to drought stress^24, 25^. However, further in-depth research underlying the molecular mechanism of drought tolerance/sensitivity in pigmented rice is still required. The high level of drought stress inhibited seed germination (%), shoot and root length and RWC. This response of growth inhibition could be attributed to low osmotic potential, decreased wall extensibility, and decreased cellular expansion^26^. In addition, while the proline content in Sahbhagi Dhan and RCPL-1-82 increased sharply with an increase in drought level via PEG stress or in pot culture, it declined in RCPL-1-185 and IR-64. Accumulation of proline under drought stress protects cells by balancing the osmotic potential of cytosol with that of vacuoles^27^; therefore imbalance in proline content may have also affected the growth and sensitivity to drought stress between the studied plants. It was observed that rice varieties with improved resilience to abiotic stress have higher rates of proline biosynthesis^28^. Elevation in superoxide anion production has been reported to be associated with increased drought stress in many plants^29^. While we observed enhanced H_2_O_2_ content in Sahbhagi Dhan and RCPL-1-82, the same declined in RCPL-1-185 and IR-64. This phenomenon could be attributed to a breakdown of H_2_O_2_ into OH and OH^−^ in RCPL-1-185 and IR-64^30^.

Drought stress induces plant defence mechanisms to protect themselves from the negative effects of oxidative stress. Plants with higher levels of induced antioxidants have better resistance and tolerance to oxidative stress^31^. ABA production also plays a significant role by enhancing its production during drought; salinity, chilling, and freezing stress^32^. High drought conditions also promoted the antioxidant enzyme activity viz. SOD, GR, MDHAR, DHAR, and CAT activity in the tolerant rice genotype (Sahbhagi Dhan and RCPL 1-82) as compared to the sensitive genotype (RCPL-1-185 and IR-64). Our results are in conformity with Sharma and Dubey^33^ and Sahoo et al.^23^. They have also reported that drought-tolerant rice varieties showed higher antioxidant enzyme activity than sensitive rice. According to Mishra and Panda^34^, increasing the expression of the antioxidant system can raise rice's tolerance to drought stress and act as a strategy against oxidative stress.

Our sub-experiment of 50% water deficit on these four rice genotypes revealed that drought stress reduced the tiller numbers, and pollen fertility, which ultimately affected the seed setting and reduction in grain yield in sensitive genotype (RCPL-1-185 and IR64), while no significant differences were observed in tolerant rice genotype (Sahbhagi Dhan and RCPL-1-82). Our results are in concurrence with Rang et al.^35^, Praba and Thangaraj^36^.

Recently, a number of drought-linked genes and transcription factors have been characterized in different plants^37, 38^. It is known that the DREB1 gene is hypersensitive to ABA signalling and a positive regulator of heat, salt, and drought tolerance in rice^39^. While P5CS is known to promote root growth and plays a significant role in plant drought resistance by enhancing proline biosynthesis^40, 41^. To find out whether the proline accumulation and antioxidant enzymes activity is regulated by the expression of respective genes, we carried out expression profiles of *DREB1*, *p5cs*, *SOD* and *WRKY* genes. PEG-mediated osmotic stress also induced expressions of drought-tolerant genes and transcription factors such as *DREB1*, *p5cs*, *SOD*, and *WRKY*. The expression profile of these genes under PEG-mediated osmotic stress was correlated with drought stress under pot culture. These results established Sahbhagi Dhan and RCPL-1-82 potent drought-tolerant rice line as compared to other studied varieties. In higher plants, WRKY TFs are expressed during environmental stresses, seed germination, seed dormancy, and other growth-related events^42, 43^. The WRKY proteins also play key functions in immune response mechanisms during mechanical damage and wounding^44^ and stress like drought stress^45^, heat shock stress^46^ etc. According to Wu et al.^47^, overexpression of *WRKY11* enhanced temperature and drought tolerance in the transgenic rice genotype. We have also found that osmotic stress enhanced the expression of *WRKY11* in the tolerant rice line; on the contrary, the expression of *WRKY114* was induced due to enhanced osmotic stress in the sensitive line. This indicates the possible role of *WRKY114* as a negative regulation of drought stress in rice. Bo et al.^48^ and Song et al.^49^ have also reported overexpression of Maize *WRKY114*, a GA-responsive gene that negatively regulated salt and drought tolerance in transgenic rice. OsWRKY114 limits rice stomatal closure, which negatively impacts the plant’s ability to withstand drought^48^. Apart from these transcription factors expression of *SOD, CAT,* and *GPX* also determines the accumulation of respective antioxidants that enhances defence mechanisms against ROS^45^. On the other hand, there was no noticeable difference in the expression of *SDR1.* While *NAC9*, *ZFP252*, *ZFP182*, and *DRAP1* expression increased in Sahbhagi Dhan and RCPL-1-82 with increasing PEG-mediated drought stress, their expression decreased in RCPL-1-185 and IR-64. Hu et al.^50^ reported the role of *OsNAC9* in increasing grain yield and enhancing root diameter in drought-tolerant rice lines. Similarly, overexpression of *ZFP252* and *ZFP182* also conferred multiple stress tolerance such as drought and salt tolerance in transgenic rice^51^. *OsDRAP1*, a gene similar to *DREB2*, is also reported to confer drought tolerance in rice by Huang et al.^52^. Our results also support the involvement of DEGs in drought tolerance or sensitivity during flowering stages in rice^53, 54^. In the current experiment of 50% water deficit under pot culture, the expression of *LOC_Os12g04500* (response regulator receiver domain-containing protein) and *LOC_Os12g26290* (alpha-DOX2), which are also known as the core of the jasmonic acid (JA) signalling pathway, was significantly increased under a prolonged drought period. Additionally, it has been shown that JA signalling genes function at key stages of drought stress^55^. Five genes, *DREB1*, *LOC_Os12g04500*, *LOC_Os02g50970, LOC_Os12g26290*, and *MYB80,* were found to have upregulated expression in drought-tolerant genotypes (Sahbhagi dhan and RCPL-1-82) and downregulated expression in drought-sensitive genotypes (IR64 and RCPL-1-185), indicating a positive relationship between these genes and drought tolerance. Pan et al.^56^ reported that MYB80 plays a key role in regulating anther development and enhancing pollen fertility. In sensitive rice genotypes, the genes *WRKY114* and *LOC_Os05g08480* (cytokinin-O-glucosyltransferase-1) showed upregulation; however, in the tolerant rice genotypes, the same genes showed downregulation. Similar results were also reported by Ahmad et al.^57^ in the young panicle of sensitive/tolerant rice genotypes.

## Conclusion

Our experiment on the effect of artificial osmotic stress via PEG-mediated and soil/pot culture stress established Sahbhagi Dhan and RCPL-1-82 as potent drought-tolerant rice genotypes as compared to other studied genotypes. We found multiple factors involved in conferring drought tolerance in rice at both metabolite and molecular levels. This study formed the preliminary base for selecting drought-tolerant rice collected from North-East India. The characterized rice genotypes may further be utilized for genome editing or rice breeding programs.

## Materials and methods

### Plant material and PEG treatment

Healthy seeds of 112 rice genotypes (Supplementary Table 4) were collected from different locations in North-East India in the year 2015–2016. Pure genotypes were grown and maintained in the greenhouse of the Department of Biotechnology, Division of Crop Science, ICAR Research Complex for NEH region, Umiam, Meghalaya, India.

In this experiment, these rice genotypes including Sahbhagi Dhan (drought-tolerant control) and IR-64 (drought-sensitive control) were subjected to osmotic stress. Healthy grains of each genotype were germinated on the individual Petri dishes. To induce osmotic stress, PEG-6000 (Merck-Schuchardt, Hohenbrunn, Germany) was used. The impact of PEG-induced osmotic stress was investigated using four treatments—T0 = 0% PEG (control condition), T1 = 10% PEG (− 0.19 MPa), T2 = 20% PEG (− 0.69 MPa), and T3 = 30% PEG (− 1.12 MPa) and applied in accordance with a completely randomised design^58^. The seed germination test was measured on the 7th DAT (Day After Treatment). After 1 week, seedlings with synchronized leaf emergence, healthy roots, and shoots were hydroponically grown (5 × Yoshida medium; diluted from 10 × Yoshida medium) in individual trays under the same PEG treatment for an additional 8 days^58^. Ten replicates were maintained for each genotype per treatment. We evaluated the water potentials prior to and following the addition of PEG to determine the osmotic stability of the nutrient solutions. Individual plant of each genotype under each treatment was taken into account for the physio-molecular analysis of root and shoot tissues, making each plant a separate experimental unit. pH was adjusted at 6.0 ± 0. 2 °C. Antioxidant activity and gene expression profiles on root and leaf tissue were conducted on the 15th DAT.

To check the expression profile of genes under PEG-mediated drought stress correlates with field experiments, a separate experiment was conducted on pot culture with a 50% water deficit^59, 60^ for four genotypes. A control (well-watered) was maintained for each genotype. Water was given on each pot (50% deficit and control) at 3–4 days intervals until seed set. Root and shoot samples were harvested on the 15th day after germination under control and drought-stressed plants. The entire experiment was conducted inside the greenhouse of the Biotechnology Department of ICAR-Umiam, maintained at 28 ± 2 °C temperature with a photoperiod of 16 h of light and 8 h of darkness.

### Percentage of seed germination and ratio of shoot to root length

The percentage of seed germination was measured after one week of treatment with PEG. The percentage of germination was calculated as follows:

$${\text{Germination}}\;(\% ) = \frac{{{\text{Number}}\;{\text{of}}\;{\text{germinated}}\;{\text{seed}}s}}{{{\text{Number}}\;{\text{of}}\;{\text{inoculated}}\;{\text{seeds}}}} \times 100.$$

Root and shoot length (cm) and root to shoot ratio were measured on 15th day after treatment.

### Estimation of relative water content (RWC)

Relative water content (RWC) was estimated in the root and shoot tissues of control and PEG-treated rice genotype on 15th DAT as described by Baldoni et al.^61^. Briefly, Relative Water Content (RWC) was determined by weighing the fresh tissues (FW), followed by floating it on deionized water for 6 h under dim light at room temperature. Fully pompous tissues were re-weighed (PW), oven dried and again weight (DW) was measured. RWC was calculated using the formula:

$${\text{RWC }} = \, \left( {{\text{FW }} - {\text{ DW}}} \right)/\left( {{\text{PW }} - {\text{ DW}}} \right) \, \times { 1}00,$$

where *FW* Fresh weight, *DW* Dry weight and *PW* Pompous weight

### Histochemical study using EVANs blue

Roots from the PEG-treated rice genotype were collected and stained with EVANs blue (Sigma-Aldrich) according to the method described by Vijayaraghavareddy et al.^62^. Briefly, 0.25 g of Evan’s blue dye was dissolved in 100 ml of 0.1 M CaCl_2_ solution at pH 5.6. Control and PEG-treated roots were excised and treated with EVANs blue for 30 min followed by rinsing with deionized water until the excess dye was removed. Qualitative estimation of EVANs staining performed under the light microscope.

### Pollen fertility test

Five mature spikelets from three panicles each of Sahbhagi dhan, RCPL-1-82, RCPL-1-185 and IR64 were collected in the morning time (6–7 am) before anthesis. The collected samples were immediately fixed in FAA solution (formaldehyde: ethanol: acetic acid in the ratio of 1:18:1). Anthers were gently crushed, stained using I_2_-KI solution and observed under an optical microscope (LEICA). Circular dark-stained pollens were considered viable pollen, while irregular light-stained/destained pollens were considered infertile.

### Estimation of total proline, hydrogen peroxide (H_2_O_2_) and ascorbate content

Total Proline was extracted according to the method of Dien et al.^28^ and estimated spectrophotometrically according to the method of Bates et al.^63^. Briefly, 100 mg root and shoot tissues were ground into fine powders using mortar and pestle. 10 ml of 80% ethanol was mixed with a 100 mg powder sample and heated in the water bath at 80 °C for 45 min. The extracted materials were filtered and used for proline estimation. 1 ml of extract was mixed with 200 µl of acid ninhydrin and 500 200 µl of glacial acetic acid in a test tube. The mixer was boiled in the water bath for 45 min followed by being cooled in an ice bath for 5 min. 1 ml of toluene was added and the mixer was vortexed. The upper layer was measured at 520 nm using toluene as a blank. Proline content was determined using the proline (Sigma-Aldrich) standard curve at 520 nm and expressed as µmol g^−1^ DW.

H_2_O_2_ concentration was measured colorimetrically as described by Jana and Choudhuri^64^.

H_2_O_2_ was extracted by homogenizing 100 mg tissues with 1 ml of phosphate buffer (pH 6.5) at low temperature followed by centrifugation at 6000×*g* for 25 min at 4 °C. The supernatant was taken in a fresh tube made up of 1 ml with phosphate buffer. H_2_O_2_ content was estimated by mixing 500 µl extract with 500 µl of 0.1% titanium sulphate in 20% H_2_SO_4_ (v/v) followed by centrifugation at 6000×*g* for 15 min at 4 °C. The yellowish colour intensity of the supernatant was measured at 410 nm using the H_2_O_2_ (Sigma-Aldrich) standard curve. H_2_O_2_ concentration was calculated using the extinction coefficient 0.28 mM^−1^ cm^−1^ and expressed as µmol g^−1^ tissue fresh weight.

Reduced ascorbate (AsA), dehydroascorbate (DHA), and total ascorbate (AsA + DHA) were extracted and quantified spectrophotometrically according to the method of Law et al.^65^.

The assay is based on AsA in an acidic solution reducing Fe3+ to Fe2+. The Fe2+ then combines with bipyridyl to produce a complex that absorbs at 525 nm and is coloured pink. The conversion of DHAs to AsA by dithiothreitol yields the measurement of total ascorbate (AsA + DHAs).

100 µl sample extract was mixed with 200 µl of 10% (w/v) trichloroacetic acid followed by standing in the ice bath for 5 min. After adding 10 µl NaOH (1 M), the mixture was centrifuged for two minutes in a Microfuge. While 100 µl supernatant was mixed with 100 µl of water and 150 mM NaH_2_PO_4_ buffer (pH 7.4). Another 100 µl supernatant was mixed with 100 µl of l 0 mM-dithiothreitol, and 100 µl of 0.5% (w/v) *N*-ethylmaleimide and kept at room temperature for 15 min. Then, 200 µl 1% (w/v) trichloroacetic acid, 200 µl 44% (v/v) H_3_PO_4_, 200 µl 4% (w/v) bipyridyl in 70% (v/v) ethanol, and 100 µl 3% (w/v) FeCl_3_ were added to each tube. Following vortex mixing, samples were incubated for 60 min at 37 °C and AaS was measured at 525 nm. 5% (w/v) m-phosphoric acid was used to develop standards for AsA and DHA in the range of 0–10 mM. DHA was calculated for each sample based on the variances between total ascorbate and AsA.

### Antioxidant enzymes assay

SOD was extracted by homogenizing 500 mg of fresh root and shoot samples in 5 ml of a buffer solution containing 100 mM potassium phosphate buffer (pH 7.5), 1.0 mM EDTA, 0.1 mM Triton X-100, and 2% polyvinylpyrrolidone (PVP). After centrifugation at 22,000×*g* for 10 min at 4 °C, the supernatant was dialyzed in cellophane membrane tubes against the cold extraction buffer. SOD was then measured by the method of Mishra and Fridovich^66^ by observing epinephrine-dependent adrenochrome formation at 475 nm in a UV–Vis spectrophotometer. The Extraction media used for GR, DHAR and MDHAR were similar to SOD. GR activity assay was estimated using the method described by Foyer and Halliwell^10^ using a reaction mixture of 80 mM Tris–HCl buffer (pH 8.5), 2.5 mM oxidized glutathione (GSSG), 1.5 mM EDTA, and 100 µl enzyme extract. The specific activity of the enzyme is expressed as nmol NADPH oxidised min^−1^ mg^−1^ protein. DHAR activity was determined according to the method described by Doulis et al.^11^ using a reaction mixture containing 90 mM sodium potassium phosphate buffer (pH 7.0), 5.0 mM reduced glutathione (GSH), 0.1 mM EDTA and 100 µl enzyme extract. The enzyme-specific activity was expressed as nmol dehydroascorbate reduced min^−1^ mg^−1^ protein. MDHAR activity was estimated by the method of Hossain et al.^12^ using a reaction mixture of 90 mM potassium phosphate buffer (pH 7.5), 0.2 mM NADH, 0.01 mM EDTA, 0.25 U ascorbate oxidase (Sigma), 0.0125% Triton X-100 and 100 µl enzyme extract. The MDHAR-specific activity was expressed as nmol NADH oxidised min^−1^ mg^−1^ protein. CAT activity was estimated according to the method of Aebi^13^ using 50 mM Tris-NaOH buffer. The rate of H_2_O_2_ degradation was determined at 240 nm with an extinction coefficient of 0.036 mM^−1^ cm^−1^. The specific activity of the enzyme was calculated as µmol H_2_O_2_ oxidised min^−1^ mg^−1^ protein. GPX was extracted in 5 ml of cold 50 mM Na-phosphate buffer (pH 7.0) and measured according to the method given by Egley et al.^14^. Specific enzyme activity was expressed as µmol H_2_O_2_ oxidized min^−1^ mg^−1^ protein.

### DNA isolation, PCR amplification and genetic data analysis

Total genomic DNA was extracted from young leaves by the CTAB (cethyltrimethylammonium bromide, Himedia) method. To evaluate the degree of genetic diversity among 112 rice accessions, a total of 62 (57 SSR and 5 RAPD) molecular markers encompassing all 12 chromosomes were utilized. These markers were specific for drought-linked QTLs (Supplementary Table 2). SSR amplification reactions were prepared for a 25 µl reaction mixture containing 2 µl of DNA (20 ng), 1 unit of Taq DNA polymerase enzyme (Promega), 2 µl of 10× buffer, 2 µl of MgCl_2_ (25 mM), 2 µl of dNTPs (2.5 mM each), 1 µl of primers (forward and reverse each), and 14.8 µl of H_2_O. SSR amplifications were performed with an initial denaturation step at 94 °C for 5 min, followed by 45 cycles of denaturation at 94 °C for 30 s, a primer annealing step at 62 °C for 45 s, an extension at 72 °C for 2 min and a final extension at 72 °C for 5 min. RAPD amplification reactions were prepared for a 25 μl reaction mixture containing 20 ng μl^−1^ template DNA, 10× buffer (NH_4_)_2_SO_4_, 2.5 mM MgCl_2_, 3.0 mM dNTPs, 0.25 μM primers, and 1 unit of Taq DNA polymerase. RAPD amplifications were performed with an initial denaturation step at 94 °C for 7 min and 30 cycles at 94 °C for 1 min, 35 °C for 1 min, and 72 °C for 2 min; the final elongation step was performed at 72 °C for 6 min. The annealing temperature varied depending on the melting temperature of each primer. Each reaction was repeated thrice. The reaction products were analyzed by electrophoresis on 1.4% agarose gels, stained with ethidium bromide, and photographed under a UV transilluminator using a digital camera with a UV filter adapter. Synthetic DNA ladder 100 bp was used as a molecular marker for the molecular weight of the bands. Each amplified band profile was defined by the presence or absence of bands at specific positions on the gel. Profiles were considered distinct if at least one polymorphic band was identified. Fragments were scored as 1 if present or 0 if absent. Allele frequency and PIC (polymorphism information content) were calculated using PowerMarker V3.25 software^67^. A model-based population structure analysis was performed using STRUCTURE software^68^. A phylogenetic tree was generated using NTSYSpc 2.10 based on the similarity index SM coefficient using 100,00 burn-in period length and 100,000 Markov Chain Monte Carlo (MCMC) repeats after burn-in^69^.

### RNA isolation, cDNA synthesis and qRT-PCR

Total RNA was isolated from the root, shoot and young panicle tissues of control and treated rice genotype using TRIzol™ RNA purification kit (Thermofisher scientific) as per manufacturer’s protocol. cDNA was synthesized using iScript cDNA synthesis kit (Biorad) following manufacturer’s protocol. Identification of target drought-responsive genes was performed based on the available literature. Transcript abundance of *DREB1* (Dehydration responsive element binding), *p5cs* (Pyrroline-5-carboxylate synthase), *WRKY11*, *WRKY114*, *rcbs* (Rubisco containing bodies), *AAO1* (Aldehyde oxidase 1), *SOD* (Superoxide dismutase), *SDR1, NAC9, ZFP252, ZFP182* and *DRAP1* (Dr1-associated polypeptide-1), *LOC_Os12g04500, LOC_Os02g50970, LOC_Os12g26290, LOC_Os05g08480, MYB80* were performed using quantitative real time PCR (qRT-PCR) and calculated as ΔCt (2^−ΔΔCT^) method^70^. The oligonucleotide primer pairs used for the amplification of drought-respective genes were designed using Primer3Plus software based on the gene sequence retrieved from NCBI and Rice Genome Database (http://rice.plantbiology.msu.edu/cgi-bin/gbrowse/rice/). Rice *Actin* gene was used to normalize the expression of drought responsive genes. A list of oligonucleotide sequences of these primers is shown in Supplementary Table 6.

### Statistical analysis

Each experiment was conducted with nine replications and represented as Mean ± SD. Tukey’s HSD test was used to determine the significant difference at p < 0.05 level using XL-STAT software, and analysis of variance was employed to test for differences.

### Ethical approval

The authors confirm that permissions or licences were obtained to collect 112 rice genotypes for the experimental purpose. All materials and methods were carried out according to the relevant guide genotype at the “Materials and methods” section.

### Supplementary Information

Supplementary Figures.

Supplementary Tables.

## Data Availability

All data supporting the findings of this study are available within the paper and within its Supplementary Materials.
