# Supplementary material for: A detailed study on genetic diversity, antioxidant machinery, and expression profile of drought-responsive genes in rice genotypes exposed to artificial osmotic stress
Source: Sci Rep. 2023 Oct 26;13:18388. doi: 10.1038/s41598-023-45661-8 (PMC10603178; doi:10.1038/s41598-023-45661-8)
Supplement: Supplementary file 1 — Supplementary Figures. [file 41598_2023_45661_MOESM1_ESM.pptx]

## Slide 1
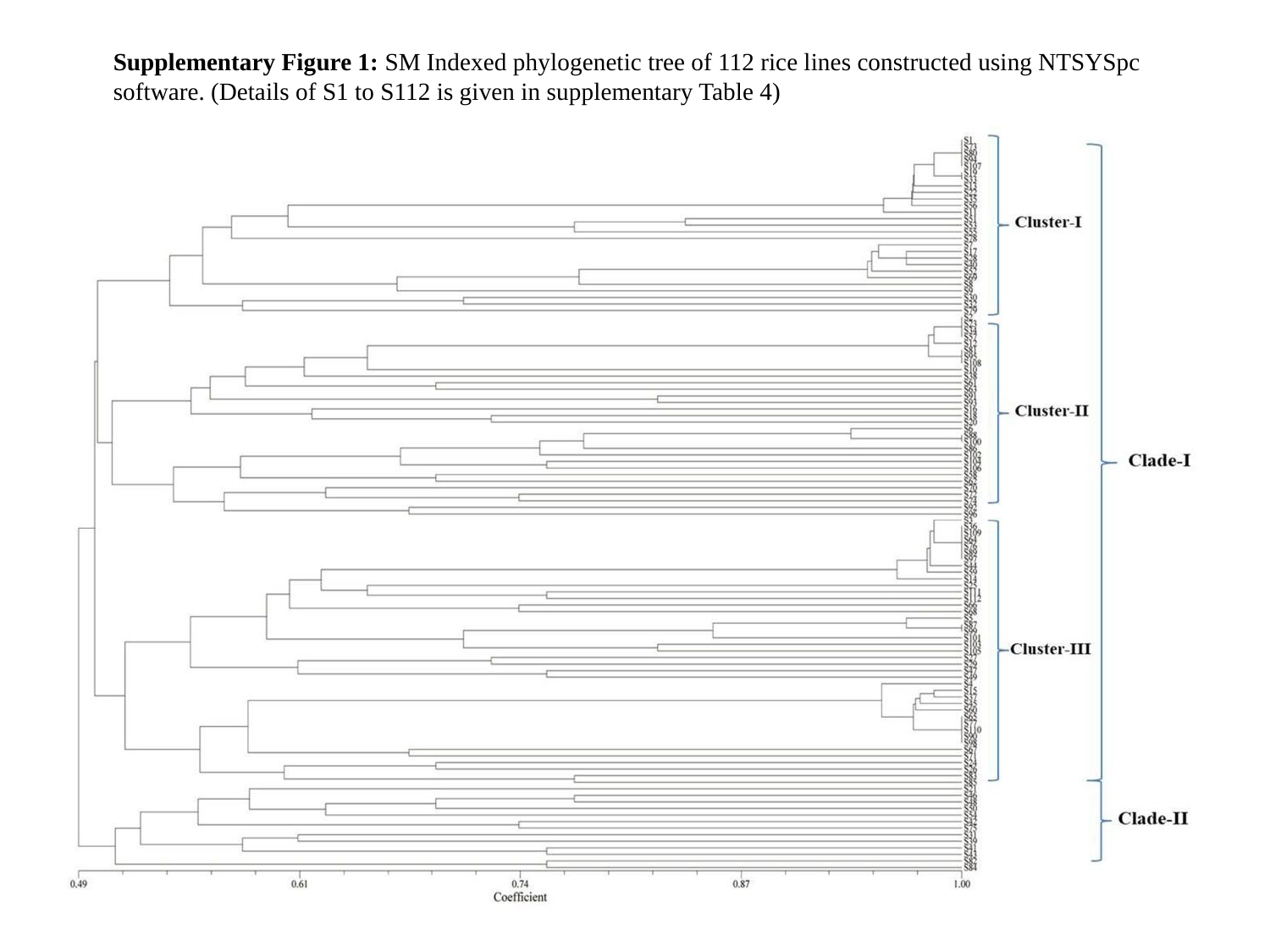

Supplementary Figure 1: SM Indexed phylogenetic tree of 112 rice lines constructed using NTSYSpc software. (Details of S1 to S112 is given in supplementary Table 4)

## Slide 2
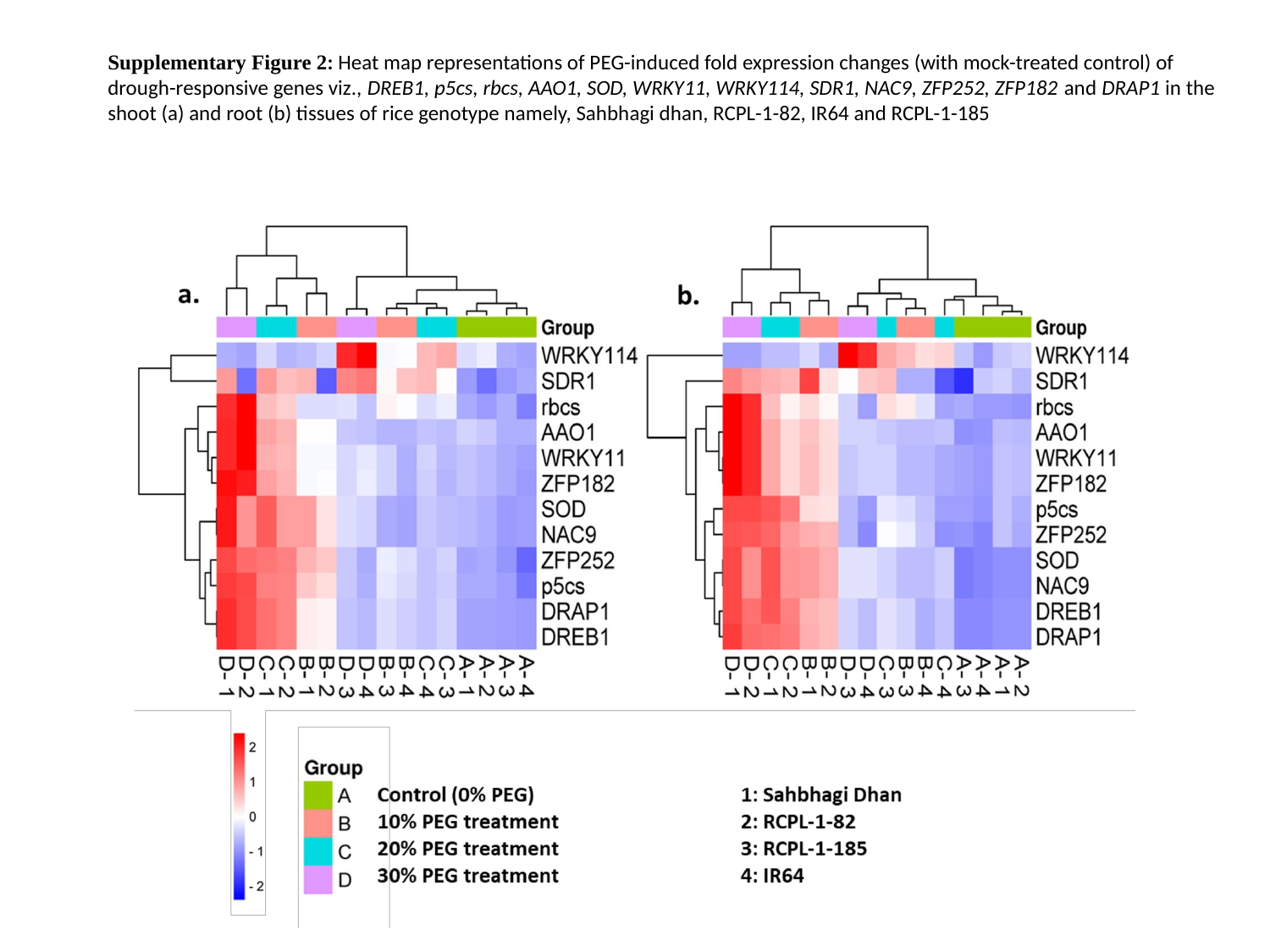

Supplementary Figure 2: Heat map representations of PEG-induced fold expression changes (with mock-treated control) of drough-responsive genes viz., DREB1, p5cs, rbcs, AAO1, SOD, WRKY11, WRKY114, SDR1, NAC9, ZFP252, ZFP182 and DRAP1 in the shoot (a) and root (b) tissues of rice genotype namely, Sahbhagi dhan, RCPL-1-82, IR64 and RCPL-1-185

## Slide 3
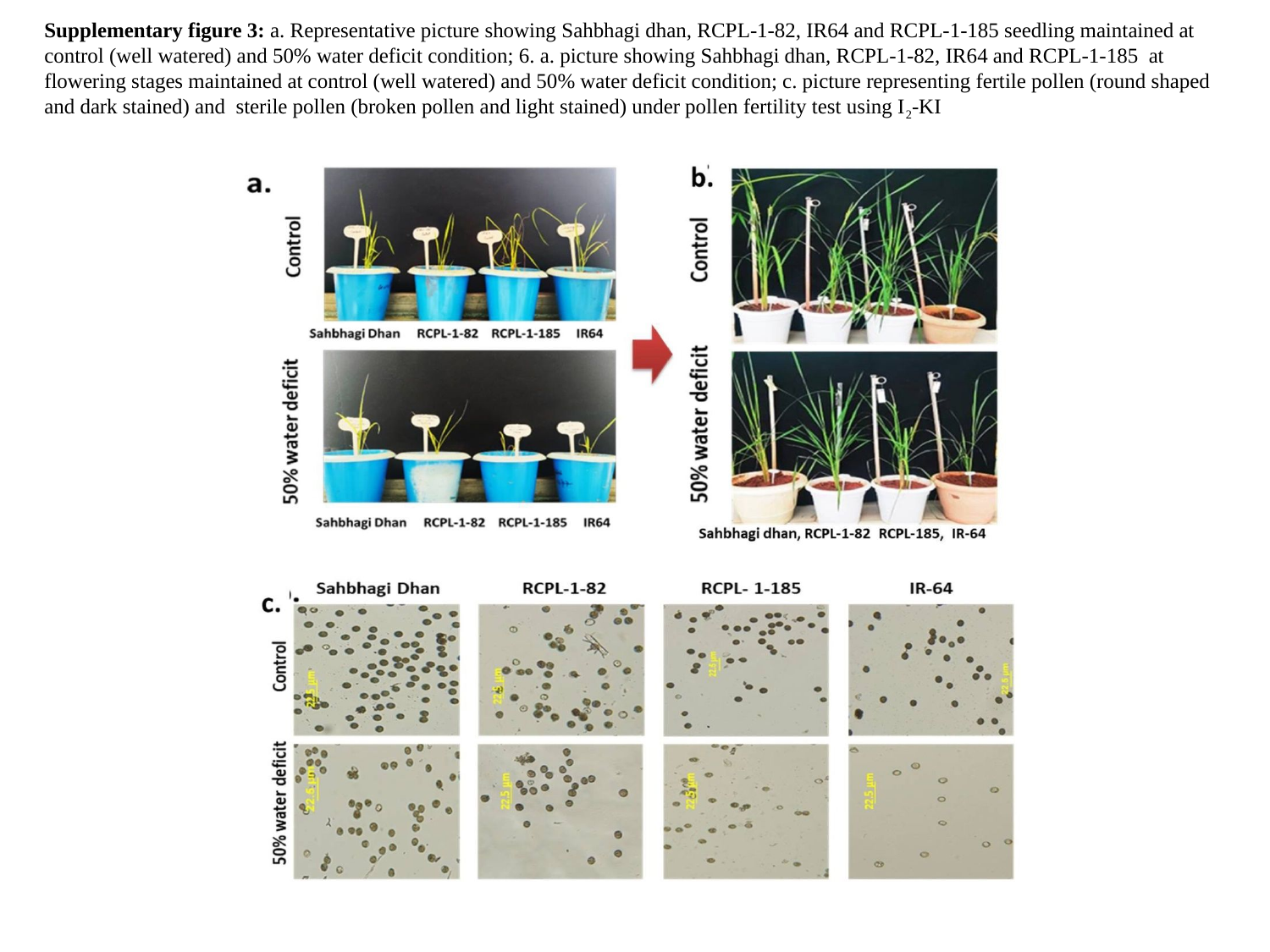

Supplementary figure 3: a. Representative picture showing Sahbhagi dhan, RCPL-1-82, IR64 and RCPL-1-185 seedling maintained at control (well watered) and 50% water deficit condition; 6. a. picture showing Sahbhagi dhan, RCPL-1-82, IR64 and RCPL-1-185 at flowering stages maintained at control (well watered) and 50% water deficit condition; c. picture representing fertile pollen (round shaped and dark stained) and sterile pollen (broken pollen and light stained) under pollen fertility test using I2-KI
