# Supplementary material for: A detailed study on genetic diversity, antioxidant machinery, and expression profile of drought-responsive genes in rice genotypes exposed to artificial osmotic stress
Source: Sci Rep. 2023 Oct 26;13:18388. doi: 10.1038/s41598-023-45661-8 (PMC10603178; doi:10.1038/s41598-023-45661-8)
Supplement: Supplementary file 2 — Supplementary Tables. [file 41598_2023_45661_MOESM2_ESM.pdf]

Supplementary table 1: Effect of PEG-mediated drought stress on Relative Water Content (RWC) in the shoots and roots of rice genotype/lines grown in NE-India; each value is representation of mean±sd, N=9 and different letters along the column are statically significant at $p<0.05$.

Rice Line	SRWC (shoot relative water content)				RRWC (root relative water content)			
	0% PEG	10% PEG	20%PEG	30% PEG	0% PEG	10% PEG	20%PEG	30% PEG
AALIDUMAJU	83.84±2.71bc	81.5±2.81cd	79.87±2.28cd	74.75±1.97de	81.25±2.08de	79.15±2.26d	77.76±1.91de	72.66±1.77de
AJUCENA	83.77±2.76bc	82.34±2.84d	78.76±2.25cd	72.61±1.91d	81.18±2.08de	79.99±2.29d	76.65±1.87de	70.52±1.72de
ANJALI	83.57±2.79bc	82.14±2.83d	77.64±2.22cd	69.61±1.83c	80.98±2.08d	79.79±2.28d	75.53±1.84d	67.52±1.65cd
Baglami	85.39±2.85bc	82.75±2.85d	81.31±2.32de	79.79±2.11ef	82.8±2.12de	80.46±2.3de	79.27±1.93e	77.17±1.92f
BANG	84.18±2.81bc	72.05±2.48ab	69.7±1.99a	68.42±1.8bc	71.59±1.84ab	69.75±1.99ab	67.59±1.65ab	66.33±1.62cd
BHALUM 1	82.93±2.76b	74.8±2.58ab	72.45±2.07b	71.17±1.87cd	74.34±1.91cb	72.45±2.07bc	70.34±1.72bc	69.08±1.68d
BHALUM 2	81.84±2.73ab	74.41±2.57ab	71.52±2.04ab	69.98±1.84c	74.25±1.9bc	72.06±2.06bc	69.41±1.69b	67.89±1.66cd
BHALUM 3	86.19±2.87c	85.95±2.96d	84.51±2.41e	82.99±2.18gf	83.6±2.14de	83.66±2.39e	82.47±2.01f	80.19±1.97g
BHALUM 4	79.56±2.65ab	75.43±2.16c	73.08±2.09bc	71.8±1.89cd	74.97±1.92bc	73.08±2.09bc	70.97±1.73bc	69.71±1.74d
BHUTMURI	81.09±2.7ab	74.96±2.58ab	72.61±2.07b	71.34±1.88cd	74.53±1.91bc	72.61±2.07bc	70.57±1.72bc	69.25±1.69d
BONG BUTAL	82.59±2.75b	74.46±2.57ab	72.11±2.06b	70.83±1.86cd	74.25±1.29bc	72.11±2.06bc	70.25±1.71bc	68.74±1.68cd
BP-2890-MR8	80.25±2.67ab	73.87±2.55ab	71.52±2.04ab	70.25±1.85cd	73.41±1.88bc	71.52±2.04b	69.41±1.69b	68.16±1.66cd
Chakhao amubi	81.69±2.72ab	71.45±2.46a	70.01±2.56ab	68.49±1.8bc	69.51±1.77a	69.14±1.97ab	67.93±1.66b	66.24±1.62dc
Chandan	82.79±2.76b	72.55±2.5ab	71.11±2.03ab	69.59±1.83c	70.42±1.18ab	70.25±2.01ab	69.22±1.68b	67.55±1.65dc
CHARANGPHOU	83.43±2.78bc	81.12±2.8cd	72.51±2.07b	63.99±1.68ab	80.84±2.07d	78.77±2.15cd	70.41±1.72bc	61.49±1.51b
COL-4	85.88±2.11c	83.57±2.88de	81.92±2.34de	76.59±2.02e	83.29±2.14de	81.22±2.03de	79.81±1.95e	74.45±1.82ef
DAGARDESHI	85.81±1.86c	83.45±2.18de	80.81±2.31d	74.45±1.96de	83.22±2.13de	81.15±2.02de	78.75±1.92de	72.36±1.76de
EPYO	85.69±2.06bc	83.38±2.54de	79.7±2.28cd	74.45±1.96de	83.15±2.13de	81.03±2.32de	77.59±1.89de	72.36±1.76de
Fullbadam	85.61±2.19bc	83.3±2.87de	79.7±2.28cd	71.45±1.88cd	83.02±2.13de	80.95±2.31de	77.59±1.89de	69.36±1.69d
GOMTIDHAN	86.03±2.17c	85.9±2.96e	83.55±2.39de	82.27±2.17gf	84.44±2.17e	83.55±2.39e	81.44±1.99ef	80.18±1.96g
GOVINDOBHOG	79.71±2.66ab	76.58±2.64c	74.23±2.12cb	72.96±1.92d	76.12±1.95c	74.23±2.12ab	72.12±1.76bc	70.87±1.73de
Hakuchung	82.79±2.76b	75.55±2.61c	74.11±2.12cb	72.59±1.91d	73.2±1.88bc	73.23±2.09ab	72.01±1.76cb	70.85±1.72de
HPR – 2558	84.31±2.81bc	82.98±2.83d	73.23±2.09bc	64.89±1.71ab	81.72±2.1de	79.65±2.28d	71.12±1.73cb	62.48±1.53bc
IORO EPYO	83.47±2.78bc	81.16±2.81cd	73.22±2.09bc	64.69±1.7ab	80.88±2.07d	78.81±2.25cd	71.11±1.73cb	62.64±1.53cb
IR-1552	83.43±2.78ab	81.12±2.18dc	72.51±2.07b	63.99±1.68ab	80.84±2.07d	78.77±2.25cd	70.48±1.72bc	61.29±1.51b
IR64	80.99±2.7ab	70.75±2.44a	69.31±1.98a	61.79±1.63a	81.45±2.09de	68.42±1.95ab	67.2±1.64ab	57.97±1.41a
IR-71524-44-1-2-8	84.52±2.82bc	82.21±2.83d	74.23±2.12bc	70.09±1.84cd	81.93±2.51de	79.86±2.28d	72.12±1.76bc	68.25±1.66cd
IR-7277-7-22-1-1	84.44±2.81bc	82.13±2.83d	74.51±2.13cb	69.05±1.82c	81.85±2.28de	79.78±2.28d	72.94±1.77bc	66.96±1.63cd
IR74052-80-1-1	84.33±2.81bc	82.02±2.83d	74.85±2.14cb	68.89±1.81bc	81.74±2.11de	79.67±2.28d	72.74±1.77bc	66.58±1.63cd
IR-78667-1-2-1-1-2	80.71±2.69ab	76.58±2.64c	74.23±2.12cb	72.96±1.92d	76.12±1.95c	74.23±2.12bc	72.12±1.76bc	70.87±1.73de
KASALATH	81.54±2.72ab	76.41±2.63c	74.06±2.12bc	72.78±1.92d	75.95±1.95bc	74.06±2.12bc	71.95±1.75bc	70.69±1.72ed
Katak tara	83.39±2.78bc	79.95±2.76cd	78.51±2.24cd	76.99±2.03e	80.48±2.07d	77.61±2.22cd	76.94±1.86de	74.19±1.83ed
Ketaki Joha	81.89±2.73ab	71.65±2.47a	70.21±2.01ab	68.69±1.81cb	79.73±2.03cd	69.32±1.98ab	68.71±1.66ab	66.46±1.62cd
KMP-34	85.58±2.85c	83.27±2.87d	79.18±2.26cd	70.95±1.87cd	82.99±2.13de	80.92±2.31de	77.07±1.88de	68.86±1.68cd
KRISHNA	85.56±2.85c	83.25±2.87d	77.54±2.22cdc	69.67±1.83c	82.97±2.13de	80.97±2.01de	75.43±1.84d	67.58±1.65cd

LUNISHREE	84.81±2.83bc	82.5±2.84d	76.93±2.2c	70.99±1.87cd	82.22±2.11de	80.15±2.29de	74.82±1.82cd	68.49±1.68cd
MAI-CHING	84.56±2.82bc	82.25±2.84d	74.24±2.12bc	71.09±1.87cd	81.97±2.1de	79.91±2.28d	72.13±1.76bc	69.25±1.68d
Megha aromatic	83.99±2.8bc	80.95±2.79cd	79.51±2.27cd	77.99±2.05ef	81.54±2.09de	78.6±2.25cd	77.84±1.89de	75.59±1.85ef
MEGHA RICE 1	83.78±2.79bc	81.47±2.81dc	79.82±2.28cd	74.49±1.96ded	81.19±2.28de	79.12±2.21d	77.71±1.91de	72.84±1.77de
MNEO	83.71±2.79bc	81.4±2.81dc	78.71±2.25cd	72.35±1.9d	81.12±1.98de	79.05±1.27d	76.86±1.87de	70.26±1.71de
N-902	83.59±2.79bc	81.28±2.8cd	77.6±2.22cd	72.35±1.9d	81.92±1.68de	78.93±2.06cd	75.49±1.84d	70.26±1.71de
NAVEEN	83.51±2.28bc	81.2±2.08cd	77.6±2.22cd	69.35±1.83c	80.92±2.17d	78.85±2.25cd	75.49±1.84d	67.26±1.64cd
NDR-97	83.48±2.18bc	81.17±2.8cd	77.08±2.2cd	65.85±1.73b	80.89±2.25d	78.82±2.35cd	74.97±1.83d	63.76±1.56cb
NEPAL RICE	83.46±2.14bc	81.15±2.8cd	75.44±2.16bc	64.57±1.7ab	80.87±2.21d	78.8±2.25cd	73.33±1.79c	62.48±1.52cb
PAIJONG	82.71±2.35b	80.4±2.77cd	74.83±2.14bc	63.89±1.68a	80.12±2.54d	78.05±2.23cd	72.72±1.77bc	61.28±1.51b
POKKALI	82.46±2.01b	80.15±2.16cd	72.14±1.99b	62.99±1.66a	79.87±1.98cd	77.08±2.22cd	70.03±1.71bc	60.89±1.49b
PR-23079-10	82.42±2.15b	80.11±2.26cd	72.13±1.56b	62.99±1.66a	79.83±1.92cd	77.76±1.92cd	70.82±1.71bc	60.19±1.41ab
PR-25679-B—9-1	82.34±2.04b	80.03±2.11cd	72.12±2.01b	62.95±1.36a	79.75±2.07cd	77.68±2.02cd	70.91±1.71bc	60.86±1.48b
PR-26850-P-J-18-6	82.23±2.14b	79.92±2.54cd	72.12±2.11b	62.79±1.55a	79.64±2.24cd	77.57±2.11cd	70.01±1.71bc	60.87±1.48b
PSB-RC2	82.21±2.01b	79.9±2.76cd	71.13±1.97ab	62.79±1.45a	79.62±2.14cd	77.55±2.01cd	69.02±1.88b	60.47±1.48ab
PURPLE RICE	81.46±2.72ab	79.15±2.73cd	71.12±2.14ab	62.65±1.65a	78.87±2.02cd	76.8±2.19cd	69.81±1.68b	60.56±1.48ab
PYNTHOR	81.37±2.71ab	79.06±2.73cd	71.12±2.01ab	62.59±1.55a	78.78±2.02cd	76.71±2.19cd	69.52±1.68b	60.65±1.48ab
RADHUNIPAGOL	82.76±2.76b	74.63±2.57ab	72.28±1.68b	71±1.87cd	74.17±1.9cb	72.28±2.07bc	70.17±1.71bc	68.91±1.68d
RANJIT	80.59±2.69ab	76.55±2.64c	75.11±2.15bc	73.59±1.94de	78±2.025cd	74.2±2.12bc	73.25±1.78c	71.45±1.74de
RCPL- 1-102	79.01±2.63ab	72.58±2.5ab	69.69±1.99a	68.15±1.79bc	72.42±1.86cb	70.23±2.01ab	67.58±1.65ab	66.06±1.61cd
RCPL- 1-108	80.55±2.69ab	72.11±2.49ab	69.22±1.98a	67.69±1.78cb	73.96±1.9cb	69.76±1.99ab	67.11±1.64ab	65.46±1.6c
RCPL- 1-112	81.04±2.71bc	71.61±2.47a	68.72±1.96a	67.18±1.77bc	75.45±1.93cb	69.26±1.98ab	66.61±1.62ab	65.09±1.59c
RCPL -1-113	83.46±2.78ab	71.02±2.45a	68.13±1.95a	66.6±1.75bc	75.87±1.95bc	68.67±1.96ab	66.02±1.61ab	64.51±1.57bc
RCPL-1-100	82.53±2.75b	70.1±2.42a	67.21±3.92a	65.67±1.73b	78.94±2.02cd	67.75±1.94a	65.11±1.59a	63.58±1.55cb
RCPL-1-101	85.06±2.84bc	82.63±2.85cd	79.74±2.28cd	78.2±2.06ef	82.47±2.11de	80.28±2.29de	77.63±1.89de	76.11±1.86ef
RCPL-1-104	84.27±2.81bc	81.83±2.82cd	78.94±2.26cd	77.41±2.04ef	81.68±2.09de	79.48±2.27d	76.83±1.87de	75.32±1.84ef
RCPL-1-105	84.48±2.82bc	83.05±2.86d	80.16±2.29d	78.62±2.07ef	81.89±2.1de	80.7±2.31de	78.05±1.91de	76.53±1.87ef
RCPL-1-107	80.17±2.67ab	73.73±2.54ab	70.84±2.02ab	69.31±1.82c	73.58±1.89cb	71.38±2.04b	68.73±1.68ab	67.22±1.64cd
RCPL-1-109	79.17±2.64ab	73.52±2.69ab	70.19±2.11ab	69.45±1.97c	73.84±1.59cb	71.38±2.14b	68.03±2.08ab	67.35±1.54cd
RCPL-1-110	85.99±2.87c	73.56±2.54ab	70.67±2.02ab	69.13±1.82c	73.4±1.88bc	71.21±2.03b	68.56±1.67ab	67.04±1.64cd
RCPL-1-115	83.53±2.78bc	81.21±2.8cd	78.21±2.23cd	76.67±2.02e	80.94±2.08d	78.75±2.25cd	76.1±1.86de	74.58±1.82ef
RCPL-1-115	81.56±2.72ab	79.13±2.73bc	76.24±2.18c	74.7±1.97de	78.97±2.02cd	76.78±2.19cd	74.13±1.81cd	72.61±1.77de
RCPL-1-117	80.31±2.68ab	77.88±2.69bc	74.99±2.14bc	73.45±1.93de	77.72±1.99cd	75.53±2.16c	72.88±1.78bc	71.36±1.74de
RCPL-1-1-185	81.69±2.72ab	70.45±2.43a	69.01±1.97a	67.49±1.78bc	78.1±2cd	71.1±2.03bb	66.9±1.63ab	60.44±1.47ab
RCPL-1-127	80.23±2.67ab	77.79±2.68bc	74.9±2.14cb	73.37±1.93de	77.64±1.99cd	75.44±2.16c	72.79±1.78bc	71.28±1.74de
RCPL-1-128	86.29±2.88c	86.05±2.97de	84.61±2.42e	83.09±2.19g	83.7±2.15de	83.74±2.39e	82.5±2.01f	81.25±1.98g
RCPL-1-13	79.22±2.64ab	76.79±2.65bc	73.9±2.11bc	72.36±1.9d	76.63±1.96c	74.44±2.13bc	71.79±1.75dc	70.27±1.71de
RCPL-1-132R	82.59±2.75v	78.75±2.72bc	77.31±2.21cd	75.79±1.99de	80±2.05d	76.49±2.18cd	75.72±1.83d	73.57±1.8e
RCPL-1-46	78.35±2.61a	75.91±2.62b	73.02±2.09bc	71.49±1.88cd	75.76±1.94cb	73.56±2.11cb	70.91±1.73bc	69.44±1.69cd
RCPL-1-46	78.27±2.61a	75.84±2.62b	72.95±2.08b	71.41±1.88cd	75.68±1.94bc	73.49±2.35bc1	70.84±1.73bc	69.32±1.69cd
RCPL-1-74	82.99±2.77b	79.35±2.74c	77.91±2.23cd	76.39±2.01e	80.4±2.06d	77.35±2.27cd	75.82±1.85d	74.73±1.81ef
RCPL-1-77	84.79±2.83cb	82.66±2.85d	80.31±2.29d	79.03±2.08ef	82.2±2.11de	80.31±2.29de	78.25±1.91de	76.94±1.88f

RCPL-1-78	82.82±2.76b	80.69±2.78cd	78.34±2.24cd	77.06±2.03ef	80.23±2.06d	78.34±2.24cd	76.23±1.86de	74.97±1.83ef
RCPL-1-82	81.57±2.72ab	79.44±2.74c	77.09±2.2cd	75.81±2de	81.98±2.1de	80.09±2.29de	79.98±1.95e	78.72±1.92fg
RCPL-1-86	81.48±2.72ab	79.35±2.74c	77±2.2cd	75.73±1.99de	78.89±2.02cd	77.35±2.2cd	74.89±1.83cd	73.64±1.83e
RCPL-1-90	80.48±2.68ab	78.35±2.7c	76±2.17c	74.72±1.97de	77.89±2cd	76.51±2.17cd	73.89±1.98c	72.63±1.77de
RCPL-1-91	79.6±2.65ab	77.47±2.67bc	75.12±2.15bc	73.85±1.94de	77.01±1.97cd	75.12±2.15c	73.01±1.78c	71.76±1.75de
RCPL-1-96	79.53±2.65ab	77.4±2.67bc	75.05±2.14bc	73.77±1.94de	76.94±1.97c	75.05±2.14c	72.94±1.78bc	71.68±1.75de
RCPL-1-97	79.39±2.65ab	77.26±2.66bc	74.91±2.14bc	73.63±1.94de	76.8±1.97c	74.91±2.14bc	72.84±1.78bc	71.54±1.74ed
RCPL-1-98	78.51±2.62a	76.38±2.63b	74.03±2.12bc	72.76±1.91d	75.92±1.95bc	74.03±2.12bc	71.92±1.75bc	70.67±1.72ed
SAHBHAGI DHAN (IR74371-70-1-1)	86.59±2.89c	85.35±2.94de	84.11±2.4e	84.09±2.21g	84.66±2.17e	82.77±2.36de	80.66±1.97ef	80.54±1.96g
SAMBA MAHSURI	78.19±2.61a	75.75±2.61b	74.31±2.12bc	72.79±1.92d	75.6±1.94bc	73.4±2.12bc	72.25±1.76bc	70.47±1.72de
SANG CHANG	81.27±2.71ab	74.14±2.56ab	71.79±2.05ab	70.51±1.86cd	73.68±1.89bc	71.79±2.05b	69.68±1.71b	68.42±1.67cd
SATABDI	78.8±2.63a	73.67±2.54ab	71.32±2.04ab	70.05±1.84cd	73.21±1.88bc	71.32±2.04b	69.21±1.69b	67.96±1.66cd
SHASHARANG	79.3±2.64ab	73.17±2.52ab	70.82±2.02ab	69.54±1.83c	72.71±1.86b	70.82±2.02ab	68.71±1.68ab	67.45±1.65cd
SHENGNYA	78.71±2.62a	72.58±2.5ab	70.23±2.01ab	68.96±1.81bc	72.12±1.85b	70.23±2.01ab	68.12±1.66ab	66.87±1.63cd
Hansa	83.79±2.79bc	71.66±2.47a	69.31±1.98a	68.03±1.79bc	73.2±1.88bc	69.31±1.98ab	67.25±1.64ab	65.94±1.61c
SKAU-390	86.32±2.88c	84.19±2.9de	81.84±2.34de	80.56±2.12f	83.73±2.15de	81.84±2.34de	79.73±1.94e	78.47±1.91fg
SLICKY RICE	85.52±2.85cb	83.39±2.88de	81.04±2.32de	79.77±2.1ef	82.93±2.13de	81.04±2.32de	78.93±1.93de	77.68±1.89f
SUKARDHAN	85.74±2.86cb	84.61±2.92de	82.26±2.35de	80.98±2.13f	83.15±2.13de	82.26±2.35de	80.15±1.95de	78.89±1.92fg
SUNDARI	79.42±2.65ab	75.29±2.6b	72.94±2.08ab	71.67±1.89cd	74.83±1.92bc	72.94±2.18bc	70.75±1.73bc	69.58±1.57d
SWARNA	80.42±2.68ab	75.29±2.6b	72.94±2.08ab	71.67±1.89cd	74.83±2.02bc	72.94±2.08bc	70.83±1.73bc	69.58±1.68d
TSAMUM FIRRI	79.25±2.64ab	75.12±2.59b	72.77±2.08ab	71.49±1.88cd	74.66±1.91bc	72.77±2.08b	70.66±1.72bc	69.84±1.69d
TSUMATSUK	81.47±2.72ab	73.34±2.53ab	70.99±2.03ab	69.71±1.83c	72.88±1.87b	70.99±2.03ab	68.88±1.68ab	67.62±1.65cd
UPR-2919	80.22±2.67ab	76.09±2.62b	73.74±2.11b	72.46±1.91d	75.63±1.94bc	73.74±2.11bc	71.63±1.75bc	70.37±1.72de
UPR-2992	81.13±2.27ab	75.7±2.61b	72.81±2.08ab	71.27±1.88cd	75.54±1.94bc	73.35±2.21bc	70.73±1.72bc	69.18±1.69d
VANDANA	81.26±2.71ab	74.82±2.58ab	71.93±2.06ab	70.4±1.85cd	74.67±1.91bc	72.47±2.07bc	69.82±1.71b	68.31±1.67cd
V-Dhan	83.69±2.79bc	80.45±2.77d	79.01±2.26cd	77.49±2.04ef	81.1±2.08de	78.1±2.23cd	76.91±1.88de	75.64±1.84ef
VIETNAM – 3	79.99±2.67ab	73.56±2.54ab	70.67±2.02ab	69.13±1.82c	73.4±1.88bc	71.21±2.03b	68.56±1.67ab	67.04±1.64cd
VIETNAM-1	84.21±2.81cb	71.78±2.48a	68.89±1.97a	67.35±1.77cb	73.62±1.89bc	69.43±1.98ab	66.78±1.63ab	65.26±1.59c
VL-31329	80.96±2.7ab	74.53±2.57b	71.64±2.05ab	70.1±1.84cd	74.37±1.91bc	72.18±2.06bc	69.53±1.71b	68.01±1.66cd
VL-31331	85.03±2.83bc	82.9±2.86d	80.55±2.3d	79.27±2.09ef	82.44±2.11ed	80.55±2.31cd	78.44±1.91de	77.18±1.88f
VPLR-1-7	84.23±2.81bc	82.1±2.83d	79.75±2.28cd	78.48±2.07ef	81.64±2.09de	79.75±2.28c	77.64±1.89dd	76.39±1.86ef
VR-14	84.45±2.82bc	83.32±2.87de	80.97±2.31d	79.69±2.1ef	81.86±2.1de	80.97±2.31cd	78.86±1.92de	77.26±1.89f
WAB-450-1-1-1-2-P41-HB	82.13±2.74ab	74.21±2.45ab	71.65±2.05ab	70.38±1.95cd	73.54±1.79bc	71.65±2.15b	69.54±1.72b	65.29±1.77c
YEMSO	81.13±2.7ab	74.57±2.55ab	71.89±2.05ab	70.54±1.85cd	73.37±1.89bc	71.45±2.05b	69.24±1.7b	62.69±1.67bc
ZAM	85.96±2.87bc	73.83±2.55ab	71.48±2.04ab	70.2±1.85cd	73.37±1.88bc	71.48±2.04b	69.37±1.69b	64.11±1.66bc

Supplementary table 2: SSR/RAPD names, allele frequency and PIC values

S. No.	SSR/RAPD marker	Chromosome	Frequency (f)	band size (bp)	PIC
1	OPA-5		0.491071	120-2635	0.758849
2	OPA-11		0.544643	58-2856	0.703364
3	OPB-10		0.285714	110-2578	0.918367
4	OPC-02		0.339286	148-2012	0.884885
5	OPD-07		0.366071	206-2566	0.865992
6	AB443	10	0.464286	235	0.784439
7	RM222	10	0.660714	128	0.563457
8	RM104	1	0.428571	221	0.816327
9	RM11	7	0.464286	100-140	0.784439
10	RM137	8	0.267857	210	0.928253
11	RM1812	11	0.321429	158	0.896684
12	RM228	10	0.517857	120	0.731824
13	RM247	12	0.303571	100	0.907844
14	RM3	6	0.258929	140-165	0.932956
15	RM304	10	0.571429	125-145	0.673469
16	RM315	1	0.5625	130	0.683594
17	RM3233	1	0.294643	165-269	0.913186
18	RM3873	1	0.473214	185-200	0.776068
19	RM431	1	0.464286	124-175	0.784439
20	RM81	7	0.375	158-178	0.859375
21	RM104	1	0.535714	210-280	0.71301
22	RM163	5	0.491071	205-270	0.758849
23	RM164	5	0.446429	152-253	0.800702
24	RM168	3	0.464286	125-190	0.784439
25	RM204	6	0.714286	145-195	0.489796

26	RM211	2	0.482143	170	0.767538
27	RM212	1	0.446429	145-175	0.800702
28	RM217	6	0.348214	120-160	0.878747
29	RM219	9	0.321429	202	0.896684
30	RM22	3	0.455357	148-180	0.79265
31	RM223	8	0.544643	165-21	0.703364
32	RM23	1	0.339286	156-254	0.884885
33	RM231	3	0.392857	195	0.845663
34	RM232	3	0.598214	221	0.64214
35	RM240	2	0.642857	231	0.586735
36	RM242	9	0.544643	245	0.703364
37	RM246	1	0.544643	116-195	0.703364
38	RM250	2	0.482143	112-175	0.767538
39	RM259	1	0.6875	251	0.527344
40	RM262	2	0.446429	180-200	0.800702
41	RM263	2	0.4375	160-180	0.808594
42	RM272	1	0.375	120	0.859375
43	RM276	6	0.598214	125	0.64214
44	RM279	2	0.473214	100-130	0.776068
45	RM289	5	0.598214	116-145	0.64214
46	RM302	1	0.678571	201	0.539541
47	RM315	1	0.491071	130-155	0.758849
48	RM316	9	0.464286	156-175	0.784439
49	RM431	1	0.410714	251	0.831314
50	RM433	8	0.339286	235	0.884885
51	RM468	3	0.419643	150-230	0.8239
52	RM486	1	0.339286	100-150	0.884885

53	RM488	1	0.392857	125	0.845663
54	RM511	12	0.4375	120	0.808594
55	RM518	4	0.607143	251	0.631378
56	RM520	3	0.464286	230	0.784439
57	RM551	4	0.383929	232	0.852599
58	RM555	2	0.464286	154	0.784439
59	RM5638	1	0.455357	168	0.79265
60	RM60	3	0.419643	210	0.8239
61	RM7	3	0.607143	235	0.631378
62	RM8115	1	0.464286	175	0.784439

Supplementary table 3: Representative code of the rice genotypes used in the supplementary figure 1.

Code	Rice line	Code	Rice line
**S1**	AALIDUMAJU	**S57**	RCPL- 1-112
**S2**	AJUCENA	**S58**	RCPL -1-113
**S3**	ANJALI	**S59**	RCPL-1-100
**S4**	Amubi (Chakhao amubi)	**S60**	RCPL-1-101
**S5**	BANG	**S61**	RCPL-1-104
**S6**	BHALUM 1	**S62**	RCPL-1-105
**S7**	BHALUM 2	**S63**	RCPL-1-107
**S8**	BHALUM 3	**S64**	RCPL-1-109
**S9**	BHALUM 4	**S65**	RCPL-1-110
**S10**	BHUTMURI	**S66**	RCPL-1-115
**S11**	BONG BUTAL	**S67**	RCPL-1-115
**S12**	BP-2890-MR8	**S68**	RCPL-1-117
**S13**	Baglami	**S69**	RCPL-1-128
**S14**	CHARANGPHOU	**S70**	RCPL-1-127
**S15**	Chandan	**S71**	RCPL-1-47
**S16**	COL-4	**S72**	RCPL-1-13
**S17**	DAGARDESHI	**S73**	RCPL-1-132R
**S18**	EPYO	**S74**	RCPL-1-485
**S19**	Fullbadam	**S75**	RCPL-1-46
**S20**	GOMTIDHAN	**S76**	RCPL-1-74
**S21**	GOVINDOBHOG	**S77**	RCPL-1-77
**S22**	Hakuchung	**S78**	RCPL-1-78
**S23**	HPR - 2558	**S79**	RCPL-1-86
**S24**	IORO EPYO	**S80**	RCPL-1-82
**S25**	IR-1552	**S81**	RCPL-1-90
**S26**	IR64	**S82**	RCPL-1-91
**S27**	IR-71524-44-1-2-8	**S83**	RCPL-1-96
**S28**	IR-7277-7-22-1-1	**S84**	RCPL-1-97
**S29**	IR-74052-80-1-1	**S85**	RCPL-1-98
**S30**	IR-78667-1-2-1-1-2	**S86**	SLICKY RICE
**S31**	KMP-34	**S87**	SAMBA MAHSURI
**S32**	Katak tara	**S88**	SANG CHANG
**S33**	KASALATH	**S89**	SATABDI
**S34**	Ketaki Joha	**S90**	SHASHARANG
**S35**	KRISHNA	**S91**	SHENGNYA
**S36**	LUNISHREE	**S92**	HANSA
**S37**	MAI-CHING	**S93**	SKAU-390
**S38**	Megha aromatic	**S94**	SAHBHAGI DHAN (IR74371-70-1-1)
**S39**	MEGHA RICE 1	**S95**	SUKARDHAN
**S40**	MNEO	**S96**	SUNDARI
**S41**	N-902	**S97**	SWARNA
**S42**	NAVEEN	**S98**	TSAMUM FIRRI

**S43**	NDR-97	**S99**	TSUMATSUK
**S44**	NEPAL RICE	**S100**	UPR-2919
**S45**	PAIJONG	**S101**	UPR-2992
**S46**	POKKALI	**S102**	VANDANA
**S47**	PR-23079-10	**S103**	VL-31331
**S48**	PR-25679-B—9-1	**S104**	VIETNAM - 3
**S49**	PR-26850-P-J-18-6	**S105**	VIETNAM-1
**S50**	PSB-RC2	**S106**	VL-31329
**S51**	PURPLE RICE	**S107**	V-Dhan
**S52**	PYNTHOR	**S108**	VPLR-1-7
**S53**	RADHUNIPAGOL	**S109**	VR-14
**S54**	RANJIT	**S110**	WAB-450-1-1-1-2-P41-HB
**S55**	RCPL- 1-102	**S111**	YEMSO
**S56**	RCPL- 1-108	**S112**	ZAM

Supplementary table 4: Rice genotypes/lines used in the present study

Rice names	Variety/Landrace	Place of collection (Institute/state or district)	IRGC Number/Accession number/ IC Number/ Other identity	Agro ecology type
AOLIDUMAJUK	Landrace	Longleng dist., Nagaland	-	Upland
AJUCENA	Variety	ICAR RCNEH Umiam, meghalaya	EC0391385	Tropical japonica
ANJALI (RR 347-166)	Released Hybrid	Gerua, Assam	-	Rainfed Upland
Amubi (Chakhao Amubi)	Landrace	Thoubal district, Manipur	-	Lowland
Bang (Ka Bang)	Landrace	East Kameng district, Arunachal Pradesh	MR39	Hill rice
Bha Lum-1 (RCPL 1-27)	Released variety from ICAR NEH, Umaim	ICAR RC NEH, Umiam, Meghalaya	-	Upland rice
Bha Lum-2 (RCPL 1-29)	Released variety ICAR NEH, Umaim	ICAR RC NEH, Umiam, Meghalaya	-	Upland rice
BHALUM 3 (RCPL1-115)	Released variety ICAR NEH, Umaim	ICAR RC NEH, Umiam, Meghalaya	-	Upland rice
BHALUM 4 (RCPL 1-116)	Released variety ICAR NEH, Umaim	ICAR RCNEH, Umiam, Meghalaya	-	Upland rice
BHUTMURI	Landrace	Hailakandi, Cachar, Assam	-	Rainfed Upland
Bongbutal	Landrace	Hailakandi, Cachar, Assam	-	Rainfed Upland and lowland
BP-2890-MR8	Landrace	ICAR RC NEH, Umiam, Meghalaya	-	Upland
Baglami	Landrace	Sonitpur Dist, Assam	-	Upland/Lowland
CHARANGPHOU	Landrace	Manipur		Upland
Chandan (CR – 898-2)	Released variety CRRI cuttack	ICAR RCNEH, Umiam, Meghalaya		Lowland
COL-4	Landrace	ICAR RCNEH, Umiam, Meghalaya	-	Upland rice
DAGARDESHI	Landrace	ICAR RCNEH, Umiam, Meghalaya	-	Lowland
EPYO	Landrace	Nagaland	-	Upland
Fullbadam	Genetic Stock	South Tripura	IC459788 AC-4423	Upland/Lowland
GOMTIDHAN	Released variety	Khawai dist. Tripura,	-	Irrigated

GOVINDOBHOG	Landrace	Kalain, Cachar, Assam	-	Medium or low -land
Hakuchuck (Tripura Hakuchuck 1)	Released variety	South Tripura	-	Lowland as well as direct seeded upland
HPR 2656	Released variety CSK Himachal Pradesh Krishi Vishvavidyalaya	West garo Hills, Meghalaya	-	Upland
IORO EPYO	Landrace	Mokokchung, Nahaland	-	Upland
IR-1552	Improved line/variety	ICAR RCNEH, Umiam, Meghalaya	-	Lowland
IR64	High yilding variety	ICAR RCNEH, Umiam, Meghalaya	-	Lowland
IR-71524-44-1-2-8	Genetic stock	ICAR RCNEH, Umiam, Meghalaya	-	Upland/Lowland
IR-7277-7-22-1-1	Genetic stock	ICAR RCNEH, Umiam, Meghalaya	-	Upland/Lowland
IR-74052-80-1-1	Genetic stock	ICAR RCNEH, Umiam, Meghalaya	-	Upland/Lowland
IR-78667-1-2-1-1-2	Genetic stock	ICAR RCNEH, Umiam, Meghalaya	-	Upland/Lowland
KMP-34	Released variety	ICAR RCNEH, Umiam, Meghalaya	-	Upland
Kataktara	Traditional cultivar/landrace	South Tripura	IRGC 49104	Upland/Lowland
KASALATH	Improved line/Variety	ICAR RCNEH, Umiam, Meghalaya	-	Upland as well as Lowland
Ketaki Joha (Aromatic)	Improved Line	Gerua, Assam	-	Low land (Shallow)
KRISHNA HAMSA	Variety	Haflong, Assam	-	Irrigated
LUNISHREE	High yielding variety	Gerua, Assam	-	Coastal Saline
MAI-CHING	Landrace	Kohima, Nagaland	-	Upland
Megha aromatic	Released Variety	ICAR RCNEH, Umiam, Meghalaya	-	Cold tolerant/Lowland
MEGHA RICE 1	Released Variety	ICAR RCNEH, Umiam, Meghalaya	-	Cold tolerant/Lowland
MNEO	Landrace	Zobawk, Mizoram	-	Upland/Midland
N-902 (Nagina 22 Mutant)	Landrace	ICAR RCNEH, Umiam, Meghalaya	-	Upland/Hill
Naveen (CR 749-20-2)	Released variety	ICAR RCNEH, Umiam, Meghalaya		Irrigated
NDR-97 (Narendra Dhan – 97)	Improved variety	Garo Hills, Meghalaya	-	Upland drought prone
NEPAL Dhan	Landrace	Jawai, Meghalaya	MR28	Hill rice

PAIJONG	Landrace	Jawai, Meghalaya	-	Lowland
POKKALI	Landrace	Sudarban area, West Bengal	IRGC 117275	Lowland/Upland
PR-23079-10	Released variety From PAU, ludhiana	Shillong, Umiam, Meghalaya	-	Upland/Lowland
PR-25679-B—9-1	Released variety From PAU, ludhiana	Shillong, Meghalaya	-	Upland/Lowland
PR-26850-P-J-18-6	Released variety From PAU, ludhiana	West Siang, Arunachal Pradesh	-	Upland/Lowland
PSB-RC2 (Nahalin)	Released variety from PSB	Wokha, Nagaland	-	Irrigated Lowland
PURPLE RICE	Landrace	Arunachal Pradesh	-	Upland/Lowland
PYNTHOR	Landrace	Jawai, Meghalaya	-	Lowland
RADHUNIPAGOL	Landrace	Karimganj, assam	-	Medium or low land
RANJIT	Improved line/variety	Hailakandi, Assam		Rain fed Medium Lands
RCPL- 1-102	Research Complex Paddy Line	ICAR RCNEH, Umiam, Meghalaya	-	Upland/Lowland
RCPL- 1-108	Research Complex Paddy Line	ICAR RCNEH, Umiam, Meghalaya	-	Upland/Lowland
RCPL- 1-112	Research Complex Paddy Line	ICAR RCNEH, Umiam, Meghalaya	-	Upland/Lowland
RCPL -1-113	Research Complex Paddy Line	ICAR RCNEH, Umiam, Meghalaya	-	Upland/Lowland
RCPL-1-100	Research Complex Paddy Line	ICAR RCNEH, Umiam, Meghalaya	-	Upland/Lowland
RCPL-1-101	Research Complex Paddy Line	ICAR RCNEH, Umiam, Meghalaya	-	Upland/Lowland
RCPL-1-104	Research Complex Paddy Line	ICAR RCNEH, Umiam, Meghalaya	-	Upland/Lowland
RCPL-1-105	Research Complex Paddy Line	ICAR RCNEH, Umiam, Meghalaya	-	Upland/Lowland
RCPL-1-107	Research Complex Paddy Line	ICAR RCNEH, Umiam, Meghalaya	-	Upland/Lowland
RCPL-1-109	Research Complex Paddy Line	ICAR RCNEH, Umiam, Meghalaya	-	Upland/Lowland
RCPL-1-110	Research Complex Paddy Line	ICAR RCNEH, Umiam, Meghalaya	-	Upland/Lowland
RCPL-1-115	Research Complex Paddy Line	ICAR RCNEH, Umiam, Meghalaya	-	Upland/Lowland

RCPL-1-115	Research Complex Paddy Line	ICAR RCNEH, Umiam, Meghalaya	-	Upland/Lowland
RCPL-1-117	Research Complex Paddy Line	ICAR RCNEH, Umiam, Meghalaya	-	Upland/Lowland
RCPL-1-128	Research Complex Paddy Line	ICAR RCNEH, Umiam, Meghalaya	-	Upland/Lowland
RCPL-1-127	Research Complex Paddy Line	ICAR RCNEH, Umiam, Meghalaya	-	Upland/Lowland
RCPL-1-47	Research Complex Paddy Line	ICAR RCNEH, Umiam, Meghalaya	-	Upland/Lowland
RCPL-1-13	Research Complex Paddy Line	ICAR RCNEH, Umiam, Meghalaya	-	Upland/Lowland
RCPL-1-132R	Research Complex Paddy Line	ICAR RCNEH, Umiam, Meghalaya	-	Upland/Lowland
RCPL-1-485	Research Complex Paddy Line	ICAR RCNEH, Umiam, Meghalaya	-	Upland/Lowland
RCPL-1-46	Research Complex Paddy Line	ICAR RCNEH, Umiam, Meghalaya	-	Upland/Lowland
RCPL-1-74	Research Complex Paddy Line	ICAR RCNEH, Umiam, Meghalaya	-	Upland/Lowland
RCPL-1-77	Research Complex Paddy Line	ICAR RCNEH, Umiam, Meghalaya	-	Upland/Lowland
RCPL-1-78	Research Complex Paddy Line	ICAR RCNEH, Umiam, Meghalaya	-	Upland/Lowland
RCPL-1-86	Research Complex Paddy Line	ICAR RCNEH, Umiam, Meghalaya	-	Upland/Lowland
RCPL-1-82	Research Complex Paddy Line	ICAR RCNEH, Umiam, Meghalaya	-	Upland/Lowland
RCPL-1-90	Research Complex Paddy Line	ICAR RCNEH, Umiam, Meghalaya	-	Upland/Lowland
RCPL-1-91	Research Complex Paddy Line	ICAR RCNEH, Umiam, Meghalaya	-	Upland/Lowland
RCPL-1-96	Research Complex Paddy Line	ICAR RCNEH, Umiam, Meghalaya	-	Upland/Lowland
RCPL-1-97	Research Complex Paddy Line	ICAR RCNEH, Umiam, Meghalaya	-	Upland/Lowland
RCPL-1-98	Research Complex Paddy Line	ICAR RCNEH, Umiam, Meghalaya	-	Upland/Lowland
STICKY RICE	Landrace	Garo hills, Meghalaya	-	Upland

Samba Mahsuri (BPT-5204)	Improved Variety	ICAR RCNEH, Umiam, Meghalaya	-	Irrigated
SANG CHANG	Landrace	ICAR RCNEH, Umiam, Meghalaya	-	Upland
Satabdi (CR 146-7027-224)	High yielding variety	Karimganj, Assam	-	Lowland
SHASHARANG	Released Variety	ICAR RCNEH, Umiam, Meghalaya	-	Lowland
SHENGNYA	Landrace	Kolashib, Mizoram	-	Upland
SHILLONG	Landrace	Umling, Meghalaya	-	Upland
SKAU-390	Landrace	West siang, Arunachal Pradesh	-	Upland
SAHBHAGI DHAN (IR74371-70-1-1)	Improved line/variety	ICAR RCNEH, Umiam, Meghalaya	-	Drought prone area
SUKARADHAN	Improved line from Himachal Pradesh	Sonamura, Tripura	-	Drought prone area
SUNDARI	Landrace	Sikkim	-	Upland/Lowland
SWARNA (MTU-7029)	Released variety	ICAR RCNEH, Umiam, Meghalaya	-	Rain-fed Lowland
TSAMUM FIRRI	Landrace	Nagaland	-	Upland
TSUMATSUK	Landrace	Zunheboto, Nagaland	-	Upland
UPR-2919 -17-3-1	Genotype	West siang, Arunachal Pradesh	-	Upland
UPR-2992-17-3-1	Genotype	West siang, Arunachal Pradesh	-	Upland
VANDANA (RR-197-962)	Improved Variety	NRRI, Cuttack	-	Rainfed Upland
VL Dhan 31331	Genotype	VPKAS, Almorah	-	Irrigated
VIETNAM – 3	Landrace	ICAR RCNEH, Umiam, Meghalaya	-	Upland/Lowland
VIETNAM-1	Landrace	ICAR RCNEH, Umiam, Meghalaya	-	Upland/Lowland
VL- Dhan 31329	Genotype	VPKAS, Almorah	-	Irrigated
Vivek Dhan-82 (IET-15473)	Released variety from ICAR- VPKAS, Almora	Arunachal Pradesh	-	Hill rice irrigated
UPLR-1-7	Landrace	Arunachal Pradesh	-	Rainfed upland
VR-14 (Virendra – CRR 347-2)	Landrace	Sikkim	-	Upland
WAB-450-1-1-1-2-P41-HB	NERICA Rice	ICAR RC NEH, Umiam, Meghalaya	-	Upland
YEMSO	Landrace	Tuensang, Nagaland	-	Upland
ZAM	Landrace	Mon dist. Nagaland	-	Upland

Supplementary table 5: Categorisation of studied rice germplasms into drought tolerant and drought sensitive based on morpho-physiological parameters under drought stress.

Groups	Class	Rice varieties	No. of genotypes
Group-I	Tolerant	Sahbhagi dhan, RCPL-1-82,baglami, Bhutmari, Bhalum-3, Bog bhutal	6
Group-II	Moderately tolerant	AALIDUMAJU, AJUCENA, ANJALI, BANG, BHALUM 1, BHALUM 2, BHALUM 4, BP-2890-MR8, CHARANGPHOU, COL-4, DAGARDESHI, Fullbadam, GOMTIDHAN, GOVINDOBHOG, Hakuchung, HPR - 2558, SAMBA MAHSURI, IR-1552, IR64, IR-71524-44-1-2-8, IR-7277-7-22-1-1, IR74052-80-1-1, IR-78667-1-2-1-1-2, KASALATH, Katak Bika, Ketaki Joha, KMP-3, KRISHNA, HANSA, Megha aromatic, MEGHA RICE 1, MNEO, N-902 , NAVEEN, NDR-97, NEPAL RICE, PAIJONG, POKKALI, PR-23079-10, PR-25679-B—9-1, PR-26850-P-J-18-6, PSB-RC2, PURPLE RICE, PYNTHOR, RADHUNIPAGOL , RANJIT, RCPL- 1-102, RCPL- 1-108, RCPL- 1-112, RCPL -1-113, RCPL-1-100, RCPL-1-10, RCPL-1-104, RCPL-1-105, RCPL-1-107, RCPL-1-109, RCPL-1-110, RCPL-1-115, RCPL-1-115, RCPL-1-117 , RCPL-1-127, RCPL-1-128, RCPL-1-13, RCPL-1-132R, RCPL-1-46, RCPL-1-46, RCPL-1-74, RCPL-1-77, RCPL-1-78, RCPL-1-86, RCPL-1-90, RCPL-1-91, RCPL-1-96, RCPL-1-97, RCPL-1-98, SANG CHANG, SATABDI, SHASHARANG, SHENGNYA, SKAU-390, SLICKY RICE, SUKARDHAN, SUNDARI, SWARNA, TSAMUM FIRRI, TSUMATSUK, UPR-2919, UPR-2992, VANDANA, V-Dhan, VIETNAM-1, VL-31329, VL-31331, VPLR-1-7, VR-14 , WAB-450-1-1-1-2-P41-HB, YEMSO, ZAM	95
Group-III	Sensitive	Chandan, Chakhao amubi, RCPL- 1-185, RCPL-112, IR64, VIETNAM - 3, Ketaki joha, IORO, EPYO, LUNISHREE, MAI-CHING,	11

Supplementary table 6: List of primers used for qRT-PCR

**Name**	**Forward Sequence (5'-3')**	**Reverse Sequence (5'-3')**	**Genebank ID (NCBI/Rice genome database)**
P5CS	TGCGAGCAGGTTAAGGAACT	GGCACAAGCCTTTCCATCTA	AY574031.1
RBCS	CGAGTTCAGCAAGGTTGGAT	AGCCGATGATACGGACAAAG	AB630180.1
SOD	GCGATATTTTCGCATCCATT	TCCAAAGGCCATTACATTCAT	LOC_Os06g05110
AAO1	TTCGCCATTTGTTCGTAA	CAGAGGAGGTTGCTCAAG	OC_Os07g18154
DREB1	GGATCAAGCAGGAGATGAGC	TACCTCGCACACCCACCT	JN561151.1
NAC9	CTGAGCTACGACGATATCCA	GAAGAGCGACGAGTAGAAGT	LOC_Os03g56580
ZFP252	CCCTTGCAAGCTCAAGAAAC	TCCACCTGTCCTCCTCCTC	AY219847.1
ZFP182	ACAAGGAGCACAGGGACAAG	GCGGTAACTAGGCGAACAAC	AY286474.1
SDR1	TGACAGCCAGGGACGAGA	TCAGCCAACCGAGAAACG	LOC_Os04g4492
WRKY11	GACGACAAGGATGAGGAGGA	GGCTGTTCTTGACTGCCTTC	BK005014.1
WRKY114	CACCCATGTTCCAAGTGACA	ATCGTCAGGGTGACCATTG	LOC_Os12g02400
DRAP1	AAGCAACGCAAGAGCTTCTC	GCAAACTGAACAGGGTCCAT	AF464902.1
Actin	TCAGGCGAATTCTCACAATG	GCTTGCTATGGATCGTGGAT	EU155408.1
LOC_Os12g04500	GGAGGTTGGAGTGAACCTGA	GAGAGCCGAACTGGTTTGAG	LOC_Os12g04500
LOC_Os02g50970	GGGCATGTTGGTGAAATACC	GAGTCGACAGGGGATACCAA	LOC_Os02g50970
LOC_Os12g26290	GCTTGTGGGACTGAAGAAGC	GCATGGTGGGGAATTATTTG	LOC_Os12g26290
Cytokinin-o-glucosyl transferase	TCATCTGGGTGACCAAGGAC	GCACTCGTTGAGGAACTGGT	LOC_Os05g08480
MYB80	CACTGGAACACGAAGCTGAA	GAGCATCTCGTCCTTGAAGC	XM_015779934.2
